# The First WHO International Standard for Adalimumab: Dual Role in Bioactivity and Therapeutic Drug Monitoring

**DOI:** 10.3389/fimmu.2021.636420

**Published:** 2021-04-15

**Authors:** Meenu Wadhwa, Chris Bird, Eleanor Atkinson, Isabelle Cludts, Peter Rigsby

**Affiliations:** ^1^ Biotherapeutics Division, National Institute for Biological Standards and Control, Medicines and Healthcare Products Regulatory Agency, Potters Bar, United Kingdom; ^2^ Analytical and Biological Sciences Division, National Institute for Biological Standards and Control, Medicines and Healthcare Products Regulatory Agency, Potters Bar, United Kingdom

**Keywords:** international standard, biosimilars, potency, clinical monitoring, unit, product life-cycle, adalimumab

## Abstract

The expanded availability of adalimumab products continues to widen patient access and reduce costs with substantial benefit to healthcare systems. However, the long-term success of these medicines is highly dependent on maintaining consistency in quality, safety and efficacy while minimizing any risk of divergence during life-cycle management. In recognition of this need and demand from global manufacturers, the World Health Organization (WHO) Expert Committee on Biological standardization established the WHO 1^st^ International standard (IS) for Adalimumab (coded 17/236) in October 2019 with a defined unitage ascribed to each of the individual bioactivities evaluated in the study namely, TNF-α binding, TNF-α neutralization, complement dependent cytotoxicity and antibody-dependent cellular cytotoxicity. For development of the IS, two candidate standards were manufactured as per WHO recommendations. Analysis of extensive datasets generated by testing of a common set of samples including the candidate standards by multiple stakeholders including regulatory agencies using their own qualified assays in a large international collaborative study showed comparable biological activity for the tested candidates for the different activities. Use of a common standard significantly decreased the variability of bioassays and improved agreement in potency estimates. Data from this study clearly supports the utility of the IS as an important tool for assuring analytical assay performance, for bioassay calibration and validation, for identifying and controlling changes in bioactivity during life-cycle management and for global harmonization of adalimumab products. In addition, in a separate multi-center study which included involvement of hospital and clinical diagnostic laboratories, the suitability of the adalimumab IS for therapeutic drug monitoring assays was examined by analysis of data from testing of a common blind coded panel of adalimumab spiked serum samples representative of the clinical scenario along with the IS and in-house standards in diverse immunoassays/platforms. Both commercially available and in-house assays that are routinely used for assessing adalimumab trough levels were included. Excellent agreement in estimates for adalimumab content in the spiked samples was observed regardless of the standard or the method with inter-laboratory variability also similar regardless of the standard employed. This data, for the first time, provides support for the extended applicability of the IS in assays in use for therapeutic drug monitoring based on the mass content of the IS. The adalimumab IS, in fulfilling clinical demand, can help toward standardizing and harmonizing clinical monitoring assays for informed clinical decisions and/or personalized treatment strategies for better patient outcomes. Collectively, a significant role for the adalimumab IS in assuring the quality, safety and efficacy of adalimumab products globally is envisaged.

## Introduction

Increased knowledge of the pathogenesis of chronic immune conditions, inflammatory disorders and some cancers has led to targeted interventions which have radically changed treatment strategies in patients with significant impact on their quality of life. Among these is the anti-TNF product class comprising Infliximab (Remicade^®^, Janssen), etanercept (Enbrel^®^, Amgen/Pfizer), adalimumab (Humira^®^, AbbVie), certolizumab pegol (Cimzia^®^, UCB) and golimumab (Simponi^®^, Janssen), all proven to be highly successful for several diseases where the pathology has been intimately linked to over production of tumor necrosis factor-alpha (TNF-α), a pleiotropic cytokine involved in the regulation of immune and inflammatory responses.

Adalimumab (Humira^®^), the world’s first fully human IgG1 therapeutic monoclonal antibody (mAb), produced using phage display technology made history when initially approved for treatment of moderate-to-severe forms of rheumatoid arthritis (RA) in 2002 and 2003 by FDA and EMA respectively (1). Humira^®^ is now indicated for use in moderate to severe polyarticular juvenile idiopathic arthritis (JIA), active psoriatic arthritis (PsA), active ankylosing spondylitis (AS), moderate to severe active adult Crohn’s disease (CD), moderate to severe active ulcerative colitis (UC), moderate to severe plaque psoriasis, uveitis and others (2–4). More recently, based on mounting evidence adalimumab is being explored for treatment of COVID-19 patients (5, 6). In terms of its function, adalimumab binds specifically to both transmembrane and soluble forms of TNF-α, the latter with high affinity preventing the interaction of TNF-α with its receptors, TNF-R1 (p55) and -R2 (p75) and modulating the signaling cascade associated with TNF-α bioactivity. The mechanism of action of adalimumab is thought to vary among diverse indications just like infliximab. In rheumatoid arthritis, adalimumab acts primarily by neutralizing soluble TNF-α, while in inflammatory bowel diseases such as Crohn’s disease and ulcerative colitis, binding to the membrane-bound form of TNF-α can trigger a range of biological effects such as alteration in levels of adhesion molecules, suppression of cytokine secretion and induction of apoptosis through reverse signaling. In addition, there can also be an interplay with Fc-mediated effector functions such as antibody dependent cellular cytotoxicity and complement mediated cytotoxicity (2, 4, 7).

While adalimumab was the third anti-TNF product to be approved for RA, the extension of the clinical use in various indications together with the ease and flexibility afforded by its subcutaneous route of administration has translated into commercial benefit. Acclaimed the bestselling product over the last three years, Humira^®^ continues to achieve global sales in excess of US$19bn (8). Such high sales and the culmination of product exclusivity in Oct’18 in Europe stimulated intense biosimilar development and approvals both in Europe and USA. Until February 2021, 12 adalimumab biosimilars (7 unique biological products) have been approved (3 have been voluntarily withdrawn due to commercial reasons) in the EU and 6 in the US (**Table 1**) with the aim of reducing costs and widening patient access (4, 7, 9–12). Unlike USA, where patents expire in 2023 (8, 13), there has been an increased uptake of biosimilars in Europe since their launch with diminishing costs to healthcare systems. In Denmark, substantial cost-reductions of ~83% have been achieved within months of shifting to biosimilars (14) while in England, savings of up to 150 million GBP a year are expected by 2021 with implementation of biosimilars in the national health service, NHS (15) against the cost of > 500 million GBP for Humira^®^ in 2017/2018. In Europe, no safety signals have been reported so far for biosimilars approved using strict criteria for biosimilarity (16–18) and product interchangeability is not a cause of any concern (19). However, product quality needs to be maintained post-approval in compliance with regulatory requirements to ensure equivalent safety and efficacy throughout the product’s lifecycle.

**Table 1:** Adalimumab products (originator and biosimilar) currently authorized in Europe and USA.

EU			Manufacturer	USA		
Tradename	INN	Approval year		Tradename	INN	Approval year
Humira ^®1^ Amgevita ^®^ Solymbic ^®2^	Adalimumab Adalimumab	Sep’03 Mar’17	AbbVie Amgen	Humira ^®1^ Amjevita ^®^	Adalimumab Adalimumab-atto	Dec’02 Sep’16
Imraldi ^®^	Adalimumab	Aug’17	Samsung Bioepis	Hadlima ^®^	Adalimumab-bwwd	July’19
Cyltezo ^®2^	Adalimumab	Nov’17	Boehringer Ingelheim	Cyltezo ^®^	Adalimumab-adbm	Aug’17
Halimatoz ^®^ Hefiya ^®^ Hyrimoz ^®^	Adalimumab	July’18	Sandoz	Hyrimoz ^®^	Adalimumab-adaz	Oct’18
Hulio ^®^	Adalimumab	Sep’18	Mylan/Fujifilm Kyowa Kirin Biologics^4^	Hulio ^®^	Adalimumab -fkjp	July’20
Idacio ^®^ Kromeya^®3^ Amsparity ^®^ Yuflyma^®^	Adalimumab Adalimumab Adalimumab	Apr’19 Feb’20 Feb’21	Fresenius Kabi Pfizer Celltrion	- Abrilada ^®^ -	- Adalimumab-afzb -	- Nov’19 -

^1^Reference product/originator product; voluntarily withdrawn in Europe in Jan’19^2^ and Dec’19^3^; ^4^authorized in Japan in June’20 as the 1^st^ biosimilar, tradename Adalimumab BS, INN - Adalimumab (Genetical Recombination) [Adalimumab Biosimilar 1].

Despite achieving clinical success, concerns over immunogenicity and loss of efficacy which are evident with other TNF inhibitors have also arisen with adalimumab (20–22). For example, in Crohn’s disease, 10–30% of patients do not respond to the initial treatment (primary failure) with anti-TNF-α mAbs and up to 46% of patients lose response over time (secondary failure), potentially due to formation of anti-drug antibodies, ADA (22) As a result, routine therapeutic drug monitoring (TDM) for measuring trough drug levels and anti-drug antibodies is being actively considered in clinical practice (23, 24). Such an approach can improve clinical decision-making, by influencing drug selection, dose, frequency of administration and potentially allowing clinicians to alter treatment strategies for patients in clinical relapse or remission. For effective treatment, it is suggested that trough levels of adalimumab need to be within a certain therapeutic window (25, 26). The American Gastroenterology Association has provided recommendations on TDM in inflammatory bowel disease, IBD (27) while in Europe, a generalized therapeutic algorithm for treatment of inflammatory diseases has been proposed (28, 29). In other indications, there is no guidance on TDM despite clinical support largely due to absence of evidence from large prospective studies (30, 31) and the lack of robust TDM data for defining the algorithm for clinical treatment. Currently, access to standardized, validated analytical methods for timely and accurate results presents a significant challenge due to different analytical techniques in use in healthcare settings (32). Although ELISAs are the commonly used platform for quantitating levels of the therapeutic, the availability of a wide range of commercial kits and in-house assays makes standardization very difficult. In the UK, the National Institute for Health and Care Excellence (NICE) has advocated the need for assay standardization (NICE diagnostics guidance [DG22] (33).

The World Health Organization (WHO) has a core role in developing norms and standards for biological medicines. This comprises elaboration of both written and measurement standards which are widely used for harmonizing practices globally. In alignment with its guidelines on biosimilar monoclonal antibodies, WHO has recognized the need for reference standards for standardizing mAbs (particularly biosimilar targets) (34–36). This has included consideration of the evolving situation in emerging markets. Unfortunately, even today, many products manufactured in these regions and approved using local regulatory pathways may not strictly adhere with the biosimilarity principles and the rigorous comparability exercise required by stringent regulatory agencies (11, 16, 17, 37), or those defined by the WHO in its guidance on similar biotherapeutic products (34, 35). Consistent with this, the National Institute for Biological Standards and Control (NIBSC) in the UK, a WHO collaborating center which produces and distributes 95% of international standards (IS) for biological medicines and vaccines, is actively engaged in the production and development of mAb reference standards, with ISs for rituximab and infliximab already established (38, 39). Such standards with defined international units are primarily intended as tools for validation of *in vitro* biological activity assays, calibration of in-house proprietary bioactivity reference standards and for harmonizing product bioactivity where possible. The use of these publicly available ISs can facilitate potency assessment not only during different phases of product development but also across products from different manufacturing processes/manufacturers and help to understand and manage any drift in bioactivity among the plethora of marketed products as they evolve post-authorization. This alignment of bioactivity is particularly important in view of the product switching that may occur not only between the originator and biosimilar product but also between biosimilar products. In some instances, however, the ISs provide an additional opportunity from the clinical perspective as they can serve as a standard for calibrating in-house standards and assays that are used routinely for measuring therapeutic drug levels e.g., diagnostic assays, commercial kits. Establishing such standards for assuring the analytical performance of the different tests for clinical monitoring can be invaluable for generating accurate and reproducible results for drug levels and would allow evidence-based decision-making for dose optimization or for treatment withdrawal/switch with better patient outcomes (28, 29).

This article describes the strategy employed for the development of the 1^st^ WHO IS for adalimumab, the third IS in the TNF product class, following WHO endorsement based on global need and priority and the results from two large, independent international collaborative studies with participation from various stakeholders (**Tables 2** and **3**). The data illustrates the suitability of a lyophilized candidate antibody preparation as an IS for *in vitro* bioactivity determination of adalimumab. In addition, for the first time, data on the extended role of the IS for assays in use for therapeutic drug monitoring is also available. This article, therefore, primarily highlights the applicability of the adalimumab IS in standardizing bioassays as well as assays for clinical monitoring of adalimumab levels.

**Table 2:** Bioassay study participants.

Aaron Farnsworth, Lori Moggy, Health Canada BGTD, 251 Sir Frederik Banting DR, Ottawa, Ontario, K1Y3L8, Canada
Michihiko Aoyama, Minoru Tada and Akiko Ishii-Watabe, National Institute of Health Sciences, 3-25-26 Tonomachi, Kawasaki-ku, Kawasaki, Kanagawa, 210-9501, Japan.
Amy Guo and Yinchuan Zhang, Department of Analytical Science, Innovent Biologics (Suzhou) Co. Ltd, 168 Dongping Street, Suzhou Industrial Park, Suzhou, 215123, China.
Ancy Nalli, Julianne Twomey and Baolin Zhang, FDA/CDER/OPQ/OBP, Bldg. 52/72, Rm. 2254, 10903 New Hampshire Avenue, Silver Spring, MD 20993, USA.
Chen Ma and Jianying Fu, Shanghai Henlius Biopharmaceuticals Co., Ltd QC lab, No.1289 Yishan Road, Shanghai, 200233, China.
Chris Bird and Parvathy Harikumar, NIBSC-MHRA, Blanche Lane, Potters Bar, Hertfordshire, EN6 3QG, UK.
Cornelius Fritsch and Paulo R. Bamert`, Novartis Pharma AG, Technical R&D, BPD, Biosimilar Bioassays,
Klybeckstrasse. 141, 4057 Basel, Switzerland.
Dietmar Eichinger and Kathrin Siegmund, Abbvie Deutschland GmbH, Knollstrasse 50, QC Biologics, Building 16, Room 406, Ludwigshafen, 67061, Germany.
Guoping Wu, Bioassay, R&D Systems, Bio-Techne, 614 McKinley Place NE, Minneapolis, MN 55413, USA.
Jean-Claude Ourlin, Direction des contrôles, ANSM, 635 Rue de la Garenne, 34740, Vendargues, France.
JongAh Joanne Lee and Junmo Yang, Samsung Bioepis Co., Ltd, 107, Cheomdan-daero, Yeonsu-gu, Incheon, 21987, Korea.
Joon Ho Eom and Jounghee Baek, Division of Advanced Therapy Product Research, National Institute of Food and Drug Safety Evaluation (NIFDS), Ministry of Food and Drug Safety(MFDS), 187, Osongsaengmyeong 2-ro, Osong-eup, Heungdeok-gu, Cheongju-si, Chungcheongbuk-do, 28159, Korea.
Julie Svennberg, Marta Löw, Magali Plas and Gaël Debauve, Bioassay development, UCB, Building T1 level2, Chemin du Foriest, Braine L’alleud, 1420, Belgium.
JunXian.Guo, Shanghai Biomabs Parmaceuticals Co.,Ltd, No 301, Libing Road, Pilot Free Trade Zone, Shanghai, 201203, China.
Keith Mortimer and Anita Carscadden, Biochemistry Section, Therapeutic Goods Administration, 136 Narrabundah Lane, Symonston, Canberra ACT 2609, Australia.
Lei Li, Zhejiang Hisun Pharmaceutical Co., Ltd. 46 Waisha Rd, Jiaojiang, Taizhou, Zhejiang, 318000, China.
Liu Yapu and Ye Hongyan, Institute of Pharmaceutical R&D, Qilu Pharmaceutical Co.Ltd, No.243 Gong Ye Bei Road, Licheng District, Jinan, Shandong, 250000, China.
Luis Meirinhos Soares and Maria João Portela, Infarmed, I.P., DCQ, Parque de Saúde – Avenida do Brasil, 53, Lisboa, 1749-004, Portugal.
Michael Tovey, Christophe Lallemand and Benoit Vallette, Biomonitor SAS, 1 mail du Professor Georges Mathé, 94800 Villejuif, France.
Omar Tounekti, Centre for Evaluation of Radiopharmaceuticals and Biotherapeutics, Health Canada, 100 Eglantine Driveway, A/L: 0602D, Tunney’s Pasture, Ottawa, Ontario, K1A 0K9 Canada
Pankaj Kalita and Sanjay Bandyopadhyay, Department of Biotechnology, Zydus Research Centre, Ahmedabad, Gujarat, 382213, India.
Sha Guo and Lan Wang, Division of Monoclonal Antibodies, National Institutes for Food and Drug Control (NIFDC), No. 31 Huatuo Road, Daxing District, Beijing, 102629, China.
Shubrata Khedkar and Mitali Samaddar, India-Biologics, United States Pharmacopeia-India (P) Ltd, Plot D6 & D8, IKP Knowledge Park, Genome Valley, Shameerpet, Hyderabad, 500078, R.R. Dist. Telangana, India.
Stuart Dunn, BioCMC LB6.1, Covance, Otley Road, Harrogate HG3 1PY, UK.
Sylvie Jorajuria, European Directorate for the Quality of Medicines and HealthCare (EDQM) Council of Europe, 7 allée Kastner, CS 30026, Strasbourg, 67081, France.
Yong Suk Yang, Celltrion Plant 2, 20, Academy-ro 51, Yeonsu-gu, Incheon, 22014, Korea.
Xujia Wang, Shanghai Junshi biosciences Co., Ltd. Room 602, No.1043 Halei road, Pudong District, Shanghai, 201203, China.

**Table 3:** Participants in adalimumab quantitation study.

Zehra Arkir and Jenny Leung, Viapath Analytics, Biochemical Sciences, St Thomas’ Hospital, 4th floor, North Wing, London SE1 7EH, UK
Raf Berghmans and Willy Mondelaers, apDia bvba, Raadsherenstraat 3, B-2300 Turnhout, Belgium
Shalini Chilakala and Kevin Carleton, Syneos Health Inc., 301D College Road East, Princeton, NJ 08540, USA
Anna Eichhorn, Corinna Berger Jana Ruppert, Immundiagnostik AG, Stubenwald-Allee 8a, 64625 Bensheim, Germany
Tom Lourens and Marianne Heij, Sanquin Diagnostics, Biologicals Laboratory, Plesmanlaan 125, 1066CX, Amsterdam, Netherlands
Daniel Nagore and Begoña Ruiz-Argüello, Progenika Biopharma S.A., Ibaizabal bidea, Parque Tecnológico Bizkaia, Ed. 504, 48160 Derio-Bizkaia, Spain
Gilles Paintaud, David Ternant, Anne-Claire Duveau and Céline Desvignes,CHRU de Tours, Laboratory Clinical Pharmacology Department, 2 Boulevard Tonnellé, 37000 Tours, France
Ermis Parussini, Guillaume Noguier and Simon Davière, Theradiag, 14 Rue Ambroise Croizat, 77183 Croissy Beaubourg, France
Mandy Perry and Rachel Nice, Royal Devon and Exeter NHS Foundation Trust, Blood Sciences, Template A2, RD&E, Barrack Road, Wonford EX2 5DW, UK
Michael Schneider and Thomas Schuster, Bühlmann Laboratories AG, Baselstrasse 55, 4124 Schönenbuch, Switzerland
Alexandra Thurston-Postle, James Pethick and Yasmin Shakil, Sandwell and West Birmingham Hospital NHS Trust, Manuals Section, Clinical Biochemistry, Sandwell General Hospital, West Bromwich B71 4HJ, UK
Thomas Van Stappen and Andrea Lennerz, R-Biopharm AG, An der neuen Bergstraße 17, D-64297 Darmstadt, Germany
Isabelle Cludts and Meenu Wadhwa, NIBSC-MHRA, Blanche Lane, Potters Bar, Hertfordshire EN6 3QG, UK
Yun Wang and Mark Heffer, Inform Diagnostics, Inc, Therapeutic Drug Monitoring Laboratory, 4207 East Cotton Centre Blvd, Phoenix, Arizona 85048, USA
Maria Willrich and Melissa Snyder, Mayo Clinic, Department of Laboratory Medicine and Pathology 200 First Street SW, Ro-Hi-2-10-PI, Rochester, MN 55905, USA
Baolin Zhang and Ancy Nalli, FDA/CDER/OPQ/OBP Bldg. 52/72, 10903 New Hampshire Avenue, Silver Spring, MD 20993, USA

## Materials and Methods

### Materials, Processing, and Characterization

Two bulk drug substance preparations of recombinant adalimumab from an originator and a biosimilar manufacturer with suitable certificates of analysis, each from a single batch were kindly donated to WHO for the purpose of developing the IS (see Acknowledgement). The materials were formulated and freeze-dried using two formulations; a) 25mM Sodium citrate tribasic dihydrate, 150mM Sodium chloride, 1% (v/v) clinical grade Human serum albumin, HSA, pH 6.5 and b) 10mM L-Histidine, 10mM L-Histidine hydrochloride monohydrate, 1% D-trehalose dihydrate, 0.01% Polysorbate-20, 1% (v/v) clinical grade HSA, pH 6.2 and tested for bioactivity in comparison with the bulk material in two different laboratories in cytotoxicity assays using WEHI-164 and L929 cell lines. Although both formulations proved to be suitable, the citrate formulation was selected for the final production fills since this provided a lyophilized preparation with marginally higher biological activity than the histidine formulation relative to the bulk material in both assays (**Supplemental Table 1**).

The production fills and lyophilization of the two candidates was performed at NIBSC using standardized procedures as specified in the WHO ECBS recommendations for International standards (40). Solutions with excipients (final compositions as shown in **Supplemental Table 2**), were prepared using nonpyrogenic water for irrigation (Baxter, Switzerland) and filtered using sterile nonpyrogenic filters (0.22μM Stericup filter system, Millipore, USA). The adalimumab content of 50 μg per ampoule was calculated from the dilution of the bulk material and assumed protein mass content provided by the manufacturer. A small batch containing a reduced amount of 40μg per ampoule was also included in the study to assess specifically the ability of the assays to distinguish a preparation with a lower amount. Optimized and controlled conditions were used for lyophilization and the glass ampoules sealed under dry nitrogen by heat fusion with storage at -20°C in the dark. Briefly, 1 ml of adalimumab solution containing approximate amounts of adalimumab (**Table 4**) was dispensed into 5 ml ampoules using an automated filling line (Bausch and Stroebel, Ilshofen, Germany) and freeze-dried in a Serail CS100 freeze-dryer (Le Coudray St Germer, France). The material was frozen over 120 minutes to -50°C and held for 6 hours at the same temperature prior to vacuum application. Primary drying was performed over 41 hours at -35°C and 100μbar vacuum followed by a ramp over 10 hours to 30°C and secondary drying for 36 hours at 30°C and 30μbar vacuum. The glass ampoules were sealed under dry nitrogen by heat fusion and stored at -20°C in the dark until shipment.

**Table 4:** Characteristics of the lyophilized preparations.

Ampoule	Study	Assumed protein content (μg)	Fill weight		Residual moisture		Headspace oxygen	
Code	Code		Mean g (n)	CV^1^%	Mean % (n)	CV^1^%	Mean % (n)	CV^1^%
17/236^2^	A, C	50	1.0082 (402)	0.175	0.195 (12)	23.03	0.36 (12)	36.00
18/124^2^	B	50	1.0082 (270)	0.180	0.402 (12)	14.41	0.28 (12)	46.02
SS711^3,4^	D	40	1.0011 (3)	0.080	0.150 (3)	34.78	0.38 (3)	7.06

^1^CV, Coefficient of Variation; n, number of estimates; ^2^The candidate preparations were expressed in CHO cells; they will be stored at -20°C at NIBSC as the custodian laboratory; ^3^small fill of 150 ampoules; ^4^This preparation was produced from the same bulk drug substance as used for 17/236 - this was included for assessing assay sensitivity or ability of the assays to detect differences and is not referred to as a candidate standard.

**Table 4** provides the characteristics of the preparations and study codes. In all instances, the specifications for WHO International standards were met. Ampoule integrity was assessed by determining residual moisture by the coulometric Karl-Fischer method (Mitsubishi CA100) and headspace oxygen content by frequency modulated spectroscopy using the Lighthouse FMS-760 Instrument (Lighthouse Instruments, LLC). No evidence of microbial contamination was found using the total viable count method.

### Participants, Study Design, and Methods

As mentioned in the Introduction, two independent collaborative studies for assessing the suitability of the IS for bioactivity and for therapeutic drug monitoring assays were organized. For confidentiality, all participant data are blind coded with a randomized laboratory number which is not related to the order of listing (**Tables 2** and **3**). Participants were encouraged to use their in-house qualified or validated methods and include routine controls and in-house reference standards where feasible. Participants were sent a study-specific protocol which provided information on the study aims and objectives, the study samples with specific instructions on their storage, reconstitution (where appropriate) and use and examples of suggested assay/plate layouts and a template for reporting of results. An independent statistical analysis of all data was performed at NIBSC.

### Bioassay Study

For this study, data was contributed by twenty-six participants from thirteen countries These comprised 12 biopharmaceutical manufacturers, 2 contract research organizations, 9 national control laboratories, 2 pharmacopoeias and 1 commercial reagent supplier (**Table 2**). All were provided with a sample pack comprising five ampoules each of samples A to C for each assay type to be performed along with 5 ampoules of the 3rd TNF-α IS (coded 12/154) for the TNF-α neutralization bioassays. Sample D containing a reduced amount of the antibody relative to samples A to C was sent to a limited number of laboratories.

Data was requested for all samples assayed concurrently on at least three separate occasions using in-house routine methods, within a suggested layout which allocated samples across 3 plates allowing for testing of replicates. Prior to performing the assay runs for the study, participants were advised however to perform pilot assay(s) using the provided samples for each of the assay type they intended to perform to ensure suitable assay conditions and establish working range for the test samples. For TNF-α neutralization bioassays, this approach allowed selection of a suitable dose of TNF-α for optimal dose response curves. Typically, most participants provided data from a total of 9 assays which included the test samples, an in-house (IH) standard (where available) in two independent dilution series on each plate using freshly reconstituted ampoules for each assay. A summary of the bioassays in the study is provided in **Table 5**.

**Table 5:** Summary of the assays performed in the collaborative study for bioactivity.

TNF-α Neutralization Assays									
Bioassay type	Cell Line		No of participants		TNF-α (IU/ml)	Assay period (hrs)		Assay readout	Readout reagent
Cytotoxicity	WEHI-164/WEHI-13VAR		7(3)		5-80	18-24		Absorbance	WST-8, CCK-8, MTS, MTT
	L929		14(8)		4-134	16-48		Fluorescence, Absorbance, Luminescence	Resazurin, Alamar Blue, Crystal Violet, CCK-8, MTS, MTT, ATP-Lite, Cell-Titer 96^®^
Apoptosis	U937		3 (2)		40-2000	2.5-4.0		Luminescence	Caspase Glo 3/7
Reporter Gene	HEK293 NF-κB-SEAP		2(1)		40	20-24		Absorbance	Quanti-Blue
	HEK293 NF-κB-Luc		5 (5)^1^		80-172	4-24		Luminescence	Steady-Glo, Dual-Glo, One-Glo
*Parentheses indicates the number of participants using in house standards in the above assays; ^1^ “ one participant used an irrelevant standard*									
**Other Cell-based Assays**									
**Bioactivity**	**Source of complement**	**Effector cells (E)**	**Target cells (T)**	**Ratio E:T**	**No of participants^2^**	**Assay type**	**Period (hrs)**	**Assay readout**	**Readout reagent**
ADCC	N/A	Jurkat-NFAT-luc-FcγRllla	CHO-mTNFα	1:10, 1:1	2^3^	Reporter gene	4 – 6/20	Luminescence	Bright-Glo,Bio-Glo
ADCC	N/A	NK92- FcγRllla	CHO-mTNFα	5:1	1	Endpoint Killing	4	Absorbance	Cytotoxicity detection kit PLUS (LDH)
ADCC	N/A	NK92- FcγRllla	3T3-mTNFα	1:1	1	Endpoint Killing	4	Luminescence	CytoTox-Glo
ADCC	N/A	NK 3.3	HEK-mTNFα	10:1	1	Endpoint Killing	1	Fluorescence	Calcein-AM
CDC	human	N/A	Jurkat-mTNFα	N/A	2	Viability	2	Absorbance	CCK-8,CellTiter-Glo
CDC	Rabbit/human	N/A	CHO-mTNFα	N/A	2	Viability	4	Luminescence	CellTiter-Glo
Cell binding	N/A	N/A	CHO-mTNFα	N/A	2	Flow cytometry	1 – 1.5	Fluorescence	Anti-human IgG (H+L) FITC/IgG Fc-PE
*^2^All participants used in-house standards (except for one performing the ^3^ADCC assay)*									
**Binding Assays**									
**Assay type**	**No of participants**	**Assay description**				**Detection reagent**		**Readout reagent**	**Assay readout**
ELISA	2^4^	Adalimumab binds to immobilized TNF-α and the bound adalimumab detected				Anti-human IgG-HRP		TMB	Absorbance
ELISA	1	Adalimumab binds to immobilized TNF-α and the bound adalimumab detected				Anti-human Kappa-HRP		TMB	Absorbance
ELISA	2	Adalimumab binds to immobilized TNF-α and the bound adalimumab detected				Anti-human IgG Fc-HRP		TMB	Absorbance
Bridging ECL	1	Adalimumab binds to Biotinylated and Sulfo-Tag labeled TNF-α and complex captured on streptavidin coated plates.				Biotin + Sulfo Tag labeled TNF-α		MSD Read buffer	Electrochemiluminescence
TR-FRET	1	Europium labeled adalimumab and Cy5 labeled TNF-α form fluorescent complex which is competitively inhibited by unlabeled adalimumab				Europium labeled adalimumab + Cy5 labeled TNF-α		N/A	Fluorescence
Biolayer Interferometry	1	Adalimumab binds to biotinylated TNF-α captured onto streptavidin biosensor.				N/A		N/A	Response binding rate (nm/s)
SPR	1	Adalimumab captured onto sensor chip immobilized with Anti Human IgG Fc, followed by concentrations of TNF-α				N/A		N/A	Response units: expressed as Equilibrium affinity constant KD (M)

All participants (except for one performing an ^4^ELISA as defined) used in-house standards.

Statistical analysis of dose-response curve data was performed using a four-parameter logistic (sigmoid curve) model (except for assays from three laboratories as specified below where a parallel line model was used)

$$y=\alpha \frac{\delta }{{1+10}^{\beta {\;(\log}_{10}{\text{x}-\log}_{10}\gamma \;)}}$$

where y denotes the assay response, x is the concentration, α is the upper asymptote, δ is the difference between upper and lower asymptotes, β is the slope factor and γ is the EC_50_ (50% effective concentration). Assay responses (absorbance, luminescence etc.) were log transformed for this analysis and it was therefore considered reasonable to combine data from all different readout formats to then derive assay validity (parallelism) criteria. Models were fitted using the R package ‘drc’ (41, 42). Parallelism (similarity) for a pair of dose-response curves was concluded by demonstrating equivalence of the parameters α, β and δ. Equivalence bound values and the methods for determining them are described in the *Results* section of this report.

Analysis of data from three laboratories (laboratories 4a - neutralization, 7 and 8 - both binding) was performed using a parallel line model due to testing of samples at fewer dilutions than other laboratories. Equivalence criteria applied to the β parameter in the sigmoid curve model analysis were used to confirm parallelism of the samples tested.

Relative potency estimates were calculated as the ratio of EC_50_ estimates in all cases where acceptable parallelism was concluded. All relative potency estimates were combined to generate unweighted geometric mean (GM) potencies for each laboratory and these laboratory means were used to calculate overall unweighted geometric mean potencies. Variability between assays and laboratories has been expressed using geometric coefficients of variation (GCV = {10^s^-1} × 100% where s is the standard deviation of the log_10_ transformed potencies).

### Study for Quantitating Adalimumab Levels

For this study, data was contributed by sixteen participants from eight countries. These included 1 contract research organization, 2 national control laboratories, 1 academic laboratory, 6 commercial kit manufacturers, 2 hospital laboratories and 4 clinical diagnostic centers (**Table 3**). All participants were provided with a sample pack comprising 4 ampoules of the lyophilized candidate preparation, Sample A (**Table 4**) and a blind-coded panel of twenty-four human serum samples prepared by spiking two pools of normal human sera (First Link and Sigma-Aldrich respectively) with either variable amounts of reconstituted candidate A or the two adalimumab preparations supplied (for use as candidates), information on amounts spiked is provided in the Results section. The samples were stored at -40°C until dispatch or use.

Prior to the study, a survey was conducted which informed on the assays in use, the assay range, sample treatment (e.g., dilution), the standard, quality control samples and the sample number easily accommodated on a single plate which helped toward study design. All participating laboratories were provided with 1 sample pack, consisting of 4 ampoules of study sample A, and adequate amounts for the serum samples for each assay type they were intending to perform. Like the bioassay study, data was requested for all samples assayed concurrently in three independent assays used routinely with inclusion of dilutions of freshly reconstituted Sample A and their own in-house (IH)/kit standard where available in each assay. Prior to performing the assay runs for the study, participants were advised to perform a pilot assay using the candidate A to ensure appropriate assay conditions and optimal dose response curves for the kit/in-house standard and candidate A. A majority of participants provided data from a total of 3 assays which included evaluation of the candidate adalimumab preparation using freshly reconstituted ampoules for each assay, the test samples and a kit/in-house (IH) standard. Information on the assays which contributed to the study is tabulated and provided in the Results section.

Statistical analysis of adalimumab levels (µg/ml) in spiked serum samples relative to sample A and kit standards or in-house standards was performed using four-parameter logistic (sigmoid curve) models. All results determined relative to sample A assumed a concentration of 50μg of adalimumab per ampoule for this standard. Estimates were combined as unweighted geometric means (GM) for each laboratory and these laboratory means were used to calculate overall unweighted geometric mean estimates. Variability between laboratories has been expressed using geometric coefficients of variation (GCV = {10^s^-1} × 100% where s is the standard deviation of the log_10_ transformed estimates). Assessment of agreement in mean estimates for each pair of laboratories was performed by calculating Lin’s concordance correlation coefficient (43, 44) with log transformed data. Calculations for this were performed using the R package ‘DescTools’ (41). A value of 1 for this coefficient indicates perfect agreement between the two laboratories.

### Reconstitution and Stability Studies

Ampoules of the candidate standard 17/236 were reconstituted and subjected to a series of freeze-thaw cycles (up to 4; n=9) or subjected separately to room temperature or 4°C for either a day or a week (n=6) and assayed concurrently against a freshly reconstituted ampoule. In addition, ampoules of the candidate standard 17/236 stored for 15 months at a range of different temperatures (45°C, 37°C, 20°C and 4°C) were tested in the L929 cytotoxicity assay alongside ampoules stored at the recommended temperature of -20°C and -70°C as baseline reference temperature. Further accelerated thermal degradation and real time stability studies for prediction of stability of the IS as per the Arrhenius equation (45) are ongoing.

## Results

The development of the IS involved multiple, sequential steps including selection of an optimal formulation, production of candidate standards, testing in two multi-center studies, data analysis and unitage assignment. Here the results of these studies which led to the recommendations to the WHO Expert Committee on Biological standardization (ECBS) and finally the establishment of the WHO IS in Oct’19 are presented.

### Preparation of Candidate Standards

WHO IS are manufactured using a strict process for lyophilization as defined in the WHO recommendations for production of reference standards (40). For maintaining stability over a long time, even decades in some cases, WHO IS are available in a lyophilized form in flame-sealed glass ampoules and contain limited amounts (μg) of the active substance unlike the high amounts (mg) in the clinical product. The characteristics of the two lyophilized candidate adalimumab preparations (coded 17/236 and 18/124), produced from generous donations of bulk drug substance from two manufacturers is given in **Table 4** and **Supplemental Table 2**. As shown, all preparations have low moisture and oxygen headspace in compliance with the WHO specifications for IS (40). A citrate formulation, which showed maximal retention of bioactivity in pilot fills comparing two different formulations in different bioassays in two laboratories and conferred stability in an accelerated thermal degradation (ATD) study was selected for lyophilization. Potency data is shown in **Supplemental Table 1**.

### Bioassay Study Design and Assays

A multi-centre, international collaborative study with 26 participants (**Table 2**) representing manufacturers, national control laboratories/regulatory agencies, contract research organizations, pharmacopoeias and commercial reagent suppliers was coordinated to evaluate the suitability of the two lyophilized candidate preparations to serve as an IS in a similar approach to other studies for WHO IS. For the study, all participants were requested to assess the activity of the candidate preparations (coded 17/236 - sample A and its duplicate sample C, 18/124 - sample B, **Table 4**), and their in-house reference standards using their own in-house qualified methods which largely comprised TNF-α neutralization assays, commonly used for lot release as well as other bioassays representative of the multiple bioactivities elicited by the antibody (**Table 5**). Details on the study design are provided in the Materials and Methods section. This practice allowed us to gain a valuable insight of the different types of cell- and non-cell based assay systems that are currently in use in different laboratories (**Table 5**) and provided information on the dose-response profile and bioactivity of the adalimumab preparations produced using different manufacturing processes, often included as in-house standards in the assay. Inclusion of an additional sample (sample D with a 20% lower adalimumab content compared with other samples), tested by a few laboratories contributed toward an increased understanding of the sensitivity of the different assays.

A summary of the bioassays is shown in **Table 5** (further details of individual participant assays is provided in **Supplemental Table 3**). As highlighted in these tables, assessment of TNF-α binding (n=8) and TNF-α neutralization(n=26) in non-cell ligand binding and cell-based assays, attributed to the Fab region of the adalimumab was a major component of the study. For binding, direct ELISAs (n=5) using immobilized TNF-α to capture adalimumab and detection with HRP-conjugated anti-IgG (Fc specific), - anti-IgG1 or - anti-kappa chain, electrochemiluminescence (ECL), fluorescence resonance energy transfer (FRET), bio-layer interferometry and surface plasmon resonance (SPR) platforms and flow cytometry based binding assays using CHO cells engineered to express non-cleavable membrane bound TNF-α (n=2) were employed. For TNF-α neutralization, three different bioassays previously used in the studies for infliximab and etanercept ISs were used (39, 46). The predominant assay (n=21) was based on the inhibition of TNF-α induced cytotoxicity of either murine fibroblast, L929 (47), or fibrosarcoma, WEHI-164 or the WEHI-13 variant cell-lines (48) followed by the reporter-gene assay (n=7) in which adalimumab inhibited TNF-stimulated activation of NF-κB transcription factor, assessed by measuring luciferase or secreted embryonic alkaline phosphatase (SEAP) activity in the human embryonic kidney cell-line, HEK-293 transfected with appropriate TNF-α responsive NFκB regulated reporter-gene constructs. Inhibition of TNF-α mediated apoptosis by measuring caspase activation in the U937 cell-line, a human histiocytic lymphoma, which exhibits properties typical of macrophages (49) was also used (n=3). Since Fc-effector function may contribute to adalimumab’s mechanism of action in some indications, complement dependent cytotoxicity (CDC) and antibody dependent cytotoxicity (ADCC) assays were included in the study. However, only a limited number of laboratories performed the CDC (n=4) and ADCC assays (n=5), possibly due to the lack of cell-lines transfected with membrane bound TNF-α. In CDC, the lysis of CHO or Jurkat T cells engineered to express a non-cleavable mutant of membrane-bound TNF-α (45) in the presence of complement was assessed. For ADCC, engineered cells (CHO/3T3/HEK-293 with membrane-bound TNF-α) served as the target. While effectors in three laboratories were natural killer cell lines e.g., the NK92 transfected with CD16a (FcγRIIIa) or the NK3.3 (instead of the conventional primary cells), which subject to CD16 engagement and activation killed target cells (50, 51), two laboratories employed surrogate ADCC assays in which reporter gene containing effectors luminesce in response to crosslinking of CD16 by adalimumab (52) in the presence of target cells (with surface-bound TNF-α).

### Bioassay Data Analysis and Dose-Response Profiles

Data received from 51 different assays (from 26 laboratories), each typically performed on three independent occasions was reviewed and an independent statistical analysis performed. An “equivalence testing” approach was adopted with curve similarity for two samples assessed using pre-defined acceptable ranges for the differences in model parameters (α, upper asymptote, δ, asymptote difference and β, slope factor). These ranges were set using neutralization data for the coded duplicates, as model parameters are expected to be equivalent for these samples in each individual assay. Absolute differences in α, log_10_β and δ parameters for the coded duplicates A & C were calculated for each plate and upper equivalence bounds set as the 95^th^ percentile of these values, taken from all laboratories performing neutralization assays. This gave upper bounds 0.078, 0.140 and 0.190 for the absolute difference in α, log_10_β and δ parameters respectively. The upper bound for log_10_β corresponds to a slope factor ratio of 1.38. For two dose-response curves to be concluded as parallel, equivalence had to be demonstrated for all three parameters (α, β and δ). The equivalence bounds applied were solely intended for use in data analysis of this study, in order to apply consistent criteria to all laboratories and assess their relative performance. The bounds should not be interpreted as suitable values for routine use in the assessment of assay validity within the collaborating laboratories. The percentage of invalid assays per lab is shown in **Supplemental Table 4** illustrating the range in relative performance of the participating laboratories using the defined equivalence criteria. Applying the global analysis to neutralization assays meant that a majority of laboratories (18 out of 23) had ≤ 25% invalid assays, indicating that this global analysis worked well and assays were of high quality, even with stringent validity parameters applied. Examination of participant data demonstrated comparable behavior and dose response profiles for all study samples although a low percentage of non-parallelism was noted between samples (sample B, coded duplicate C or in-house standard) in a minority of assays across the study. Importantly, the resemblance in behavior across most assays regardless of the assay type or the samples including in-house standards (except those that were irrelevant) confirmed the suitability of the candidates as bioassay standards for calibration of different adalimumab products.

### Potency Estimates Relative to In-House Reference Standards or Sample A

**Figure 1:**
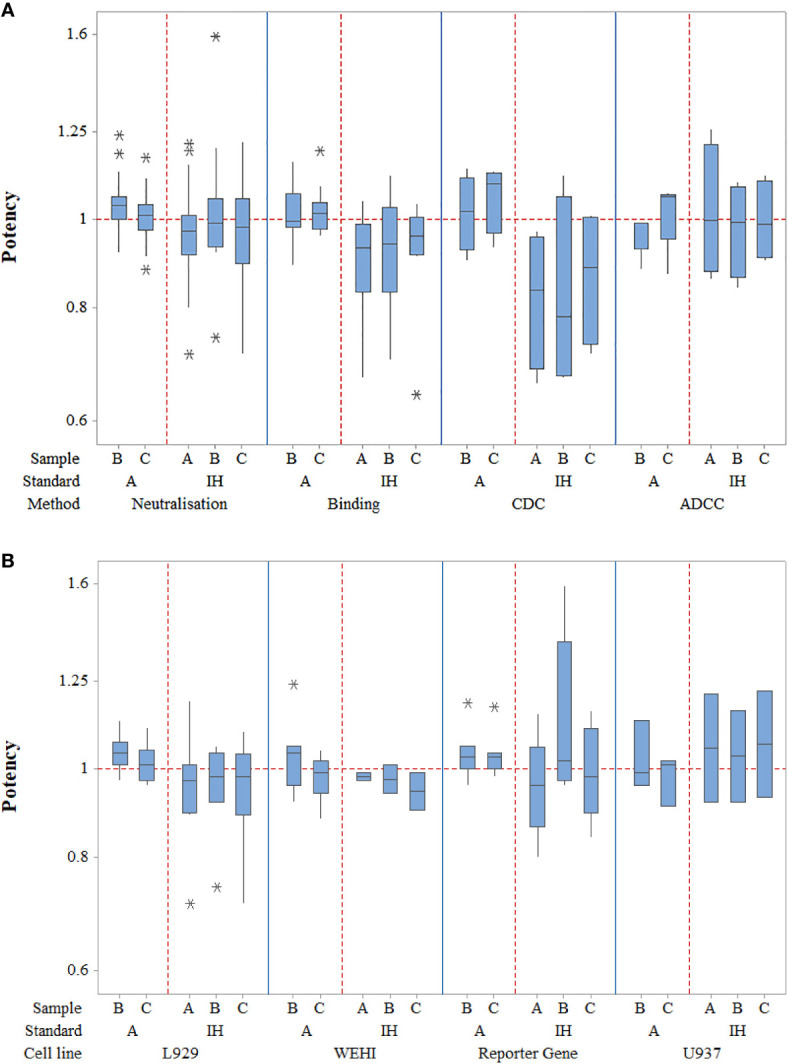
Laboratory geometric mean relative potency estimates for all different assay types **(A)** as well as for the different TNF-α cell-based neutralization assays **(B)**. Boxes represent the interquartile range and the line shows the median. The bars represent the range and * shows outliers defined as 1.5 times the interquartile range.

Potency estimates calculated relative to candidate standard sample A or relative to in-house reference standards where available (adalimumab manufactured in-house, n=9; Humira batch, n=7; research grade anti-TNF antibody, n=1; an irrelevant anti-TNF, n=1) for different assays from individual laboratories are summarized in **Supplemental Tables 5**–**7**. An overall summary of potency for each assay type is shown in **Table 6** and boxplots of laboratory geometric mean (GM) relative potencies are shown in **Figure 1**.

**Table 6:** Overall geometric mean relative potency estimates for all assays contributed to the study.

Method	Sample	Potencies relative to sample A					Potencies relative to in-house reference*				
		GM	LCL	UCL	GCV	N	GM	LCL	UCL	GCV	N
Neutralization (all)	A	–	–	–	–	–	0.97	0.90	1.03	14.03	17
	B	1.04	1.01	1.06	6.43	32	1.01	0.93	1.10	17.30	16
	C	1.01	0.99	1.03	5.61	32	0.97	0.91	1.04	13.60	17
	D	0.86	0.81	0.91	9.28	11	0.73	0.48	1.11	30.55	4
Neutralization (WEHI)	A	–	–	–	–	–	0.98	n/a	n/a	n/a	2
	B	1.03	0.94	1.13	10.08	7	0.97	n/a	n/a	n/a	2
	C	0.98	0.93	1.03	6.08	7	0.94	n/a	n/a	n/a	2
	D	0.88	n/a	n/a	n/a	2	0.92	n/a	n/a	n/a	1
Neutralization (L929)	A	–	–	–	–	–	0.95	0.84	1.07	15.88	8
	B	1.04	1.01	1.06	4.33	15	0.95	0.85	1.06	12.59	7
	C	1.02	0.99	1.04	4.67	15	0.94	0.84	1.06	14.83	8
	D	0.83	0.79	0.86	3.26	5	0.81	n/a	n/a	n/a	1
Neutralization (Reporter Gene)	A	–	–	–	–	–	0.96	0.81	1.12	13.83	5
	B	1.04	0.98	1.11	6.58	7	1.13	0.87	1.46	23.30	5
	C	1.04	0.98	1.09	5.85	7	0.99	0.86	1.15	12.65	5
	D	0.88	0.71	1.09	14.48	4	0.62	n/a	n/a	n/a	2
Neutralization (U937)	A	–	–	–	–	–	1.05	n/a	n/a	n/a	2
	B	1.02	0.83	1.26	8.88	3	1.03	n/a	n/a	n/a	2
	C	0.98	0.84	1.14	6.35	3	1.07	n/a	n/a	n/a	2
	D	–	–	–	–	–	–	–	–	–	–
ADCC	A	–	–	–	–	–	1.02	0.78	1.34	18.10	4
	B	0.97	0.90	1.03	5.44	5	0.98	0.80	1.19	13.03	4
	C	1.02	0.91	1.14	9.32	5	1.00	0.85	1.17	10.84	4
	D	0.79	n/a	n/a	n/a	1	0.91	n/a	n/a	n/a	1
Binding	A	–	–	–	–	–	0.90	0.80	1.00	15.22	9
	B	1.01	0.96	1.07	7.36	10	0.92	0.82	1.03	16.03	9
	C	1.02	0.98	1.07	6.91	10	0.93	0.83	1.03	15.53	9
	D	0.83	0.72	0.95	9.10	4	0.75	0.71	0.80	2.46	3
CDC	A	–	–	–	–	–	0.82	0.62	1.08	19.13	4
	B	1.02	0.87	1.19	10.34	4	0.83	0.56	1.22	27.56	4
	C	1.06	0.92	1.22	9.05	4	0.87	0.66	1.14	19.11	4
	D	0.73	n/a	n/a	n/a	1	0.69	n/a	n/a	n/a	1

GM, Geometric Mean; LCL, Lower 95% Confidence Limit; UCL, Upper 95% Confidence Limit; GCV, Between-laboratory Geometric Coefficient of Variation (%); N, Number of laboratories used in calculation of GM and GCV; *Lab 4b excluded (used a different TNF antagonist as IH reference standard; n/a, not calculated as N<3.

In terms of neutralization activity, potency estimates for candidates A and its coded duplicate, C were identical and determined as 0.97 relative to the in-house standards; the inter-laboratory variability, expressed as % geometric coefficient of variation (GCV) was also similar at 14.03 and 13.60 respectively. For sample B, the potency was also very close at 1.01 with a GCV of 17.30%. Use of candidate A as a standard for determining relative potencies gave estimates of 1.04 and 1.01 for B and C, which again were very similar to those seen with in-house standards but the inter-laboratory variability was greatly reduced (6.43% and 5.61% respectively) relative to A in comparison with the higher values (17.30% and 13.60% respectively) for in-house standards. Intra-laboratory variability for the potencies of samples B and C relative to A ranged from 2.27% to 32.02% in neutralization assays, with a median value of 7.83% and the majority (63%) of values were less than 10% (87% less than 20%), demonstrating generally good intermediate precision in participating laboratories (n=26). Overall, the levels of variability in neutralization assays were comparable to those seen in binding assays regardless of the standard used. For binding assays, intra-laboratory GCV values ranged from 0.61% to 32.32% relative to sample A and 4.48% to 28.08% in comparison with in-house standards. Inter-laboratory GCV values for samples B and C were 7.36% and 6.91% versus A and 16.03% and 15.53% respectively versus in-house standards. All neutralization assays were fairly comparable in terms of their GCVs (<11%) when a common standard, A is used; the lowest inter-laboratory variability was observed in the L929 cytotoxicity assay with GCV of 4.33% and 4.67% for B and C relative to candidate A and 12.59% and 14.83% when the in-house standards were used. Since there were fewer valid assays using in-house standards for laboratories undertaking WEHI-164 and U937 assays, improvement of inter-laboratory GCV with use of sample A could not be determined. As for other assays, the inter-laboratory GCV for B and C in reporter gene assays was considerably reduced relative to A in comparison with in-house standards (6.58% and 5.85% vs. 23.30% and 12.65%). Overall, a higher level of inter-laboratory variability for potencies relative to in-house standards compared with potencies relative to A was evident.

Potency estimates using CDC and ADCC assays were consistent with values from neutralization and binding assays. Intra-laboratory variability was noted to be similar for CDC assays (2.71% to 36.07%) with a narrower range of %GCV for ADCC assays (6.9% to 23.84%) and a wider range for binding assays (0.61% to 32.32%) when assessed relative to sample A. However, when in-house standards were used, the intra-lab variability range was wider for ADCC assays compared with other assays. The inter-laboratory variability was higher for CDC as opposed to ADCC or binding assays regardless of the standard.

To conclude, the study data showed that the use of sample A as a reference standard to calculate the relative potency of sample B allowed a close agreement between laboratories for each of the bioactivities tested in comparison with in-house standards.

### Potency Estimates of D Relative to Sample A

In laboratories which tested Sample D (n=11) in neutralization assays, the overall GM potency was 0.86 relative to A with a GCV of 9.28%, with potency less than 0.90 in all but two cases (0.94 and 1.07) consistent with the expected theoretical value (**Table 6**, **Supplemental Table 5**). Similar observations were noted in binding assays (range 0.77 to 0.94, n=4) and in the ADCC and CDC assays (**Supplemental Tables 6** and **7**) indicating that the assays where this sample was tested were adequately sensitive in detecting lower activity associated with reduced content.

### Estimates of ED50 Derived From Neutralization Assays

To assess the inhibitory effect of adalimumab in TNF-α neutralization assays, geometric mean ED50 estimates were derived for each sample (**Supplemental Table 8**), these values varied between different laboratories and assay methods and no clear relationship with the TNF-α dose was observed. A summary of ED50 estimates for L929 assays from selected laboratories using a fixed TNF-α concentration of 20 IU is given in **Table 7**; the geometric mean ED50 value was used in the following equation:

$$Amount\;of\;adalimumab\;\left(IU\right)\;inhibiting\;a\;fixed\;amount\;of\;TNF-\alpha \left(IU\right)=\frac{potency\;of\;preparation\;\left(IU\right)\;x\;ED50\;\left(ng\right)}{Assumed\;mass\;content\;\left(ng\right)}$$

**Table 7:** Summary of ED50 estimates (ng) for selected L929 neutralization assays using a fixed amount of TNF-α (20IU).

Sample	GM	LCL	UCL	GCV	N
A	8.47	5.92	12.12	33.49	5
B	7.90	5.62	11.12	31.63	5
C	8.25	5.64	12.06	35.76	5
D	9.67	n/a	n/a	n/a	2
IH	5.94	n/a	n/a	n/a	2

GM, Geometric Mean; LCL and UCL, Lower and Upper 95% confidence limits; GCV, Geometric Coefficient of Variation (%); N, Number of estimates used in calculation of GM and GCV; n/a, not calculated as N<3.

Therefore, based on data from five laboratories (**Table 7**), 0.085 IU of candidate A, (code 17/236) inhibits the cytotoxic effect of 20 IU of TNF-α IS (code 12/154) in an L929 cytotoxicity assay. The arbitrary unitage of 500 IU for the adalimumab candidate A coded 17/236 was used to derive the inhibitory activity.

### Stability Studies

ISs are intended to be long-lasting stable preparations suitable for global distribution in their role as ‘higher order’ standards. Formulation and process development is therefore optimized to fulfill this requirement while preserving bioactivity for the standard’s intended use in supporting calibration and/or stability of secondary standards (manufacturer, regional, pharmacopoeia) in use for potency assays for clinical products world-wide. Post-reconstitution studies showed retention of potency after 1 week of storage at either 4˚C or 20˚C or after repeated freeze-thaw cycles (**Supplemental Tables 9** and **10**). ATD studies over 15 months indicated that the bioactivity of the candidate preparation 17/236 did not deteriorate (**Supplemental Table 11**) despite storage at elevated temperatures supporting its utility as an IS. With no loss in activity seen at high temperatures, no predicted loss in activity could be calculated. Further real time stability studies will be undertaken to monitor and predict potential loss of activity over time.

### Study Design and Assays for Assessing Adalimumab Levels

A separate study was designed to assess the suitability of a candidate adalimumab preparation to serve as the 1^st^ WHO IS for assays measuring adalimumab levels in the clinical setting. A survey conducted prior to the study informed on dose range, sample dilution and matrix, the standard, quality control (QC) samples of the assays and facilitated study design. Sixteen participants from eight countries, listed in **Table 3**, representing national control laboratories, contract research organizations, commercial kit manufacturers, academia, hospital laboratories and clinical diagnostic centers contributed data. This data included results from testing of a panel of twenty-four human serum samples spiked with different amounts of adalimumab to assess the suitability of the IS in measuring levels in a serum matrix (i.e. conditions reflecting the clinical scenario) and also for evaluating assay analytical performance in instances where the same assay type/kit was used in multiple laboratories. All participants tested the blind-coded panel along with the candidate preparation, Sample A (**Table 4**) and the in-house (IH)/kit standard (and QC samples) where available concurrently on the same plate, in three independent assays, as per the study protocol after performing a pilot assay to ensure appropriate assay conditions and optimal dose response curves for assay standards.

A summary of the assay methods used by the study participants, all measuring free adalimumab is given in **Table 8**. As expected, ELISAs were the predominant assay, performed by twelve participants. A majority of the ELISAs were commercial kits (n=10) but in-house assays were also performed (n=2). ELISA formats varied (53–55). In some cases, other anti-TNF-α therapeutics could also be detected, however, most were specific for adalimumab. Adalimumab was captured either by immobilization of TNF-α or an anti-adalimumab antibody, both used in multiple laboratories and detected using different secondary antibodies which were mainly either anti-adalimumab antibodies or anti-human IgG antibodies. Rapid point-of-care devices based on the lateral flow immunoassay (LFI) technology (56) were used in two laboratories. In these assays, capillary action allows interaction between the adalimumab and TNF-α conjugated to gold colloid. This complex is then captured by immobilized anti-adalimumab antibody providing a visual response and a measurable read-out. ECL assays employing the stable sulfotag label that emits light on voltage stimulation, in an appropriate chemical environment were also used (n=2) though the format varied with one participant adopting the sequential ELISA-like approach (with immobilized TNF-α, followed by sample incubation and finally sulfotag-labeled anti-human IgG kappa light chain for detection) while the other using solution phase (samples were incubated simultaneously with biotin- and sulfotag-labeled TNF-α, transferred onto a streptavidin plate) for detecting antigen-antibody complexes by measuring the ECL signals (57).

**Table 8:** Brief details of assays contributed for assessing adalimumab levels.

Lab code	Assay platform	Assay description	Assay Standard(s)	Read-out	Specific
1T, 10T, 11T	ELISA (C)	Plates coated with TNF-α, adalimumab captured, detected with biotin anti-human IgG followed by HRP-streptavidin	Kit & IH (1T) Kit (10T, 11T)	OD	no
1T	ELISA (C)	Plates coated with TNF-α, adalimumab captured, detected with biotin anti-adalimumab followed by HRP-streptavidin	Kit & IH	OD	yes
2T	ELISA (C)	Plates coated with anti-adalimumab, adalimumab captured, detected with HRP anti-adalimumab.	Kit	OD	yes
3T,12T-14T	ELISA (C)	Plates coated with anti-adalimumab, adalimumab captured, detected with HRP antibody.	Kit & IH (3T) Kit (12T-14T)	OD	yes
4T	ELISA (C)	Plates coated with TNF-α, adalimumab captured, detected with HRP-anti-adalimumab	Kit & IH	OD	yes
5T	ELISA (C)	Plates coated with anti-adalimumab, adalimumab captured, detected with HRP conjugate.	kit	OD	yes
6T	ELISA (IH)	Plates coated with TNF-α, adalimumab captured, detected with HRP anti-human IgG	IH	OD	no
9T	ELISA (IH)	Plates coated with anti-TNF-α, followed by capturing of adalimumab using TNF-α and detection with biotin anti-adalimumab and HRP streptavidin.	IH	OD	yes
7T	ECL (IH)	Plates coated with TNFα, adalimumab captured, then addition of sulfotag anti-human kappa light chain.	IH	counts	no
8T	ECL (IH)	Samples incubated with biotinylated TNFα and sulfotag TNFα, transferred to streptavidin plate.	IH	counts	no
15T	LFI (C)	Adalimumab is detected *via* the formation of a ‘sandwich’ with TNF-α and an anti-adalimumab	Kit (pre-defined) & IH	OD	yes
16T	LFI (C)	Adalimumab detected *via* the formation of a ‘sandwich’ with TNF-α and an anti-adalimumab	Kit (pre-defined) & IH	OD	yes

All standards are Humira^®^ based; Kit standards only, participants using a purchased commercial kit; IH-In-house standards only, participants using an in-house method; Kit & IH, commercial kit manufacturers; LFI, lateral flow immunoassay; ECL, electrochemiluminescence, Parentheses indicates commercial C or in-house IH assay.

### Data on Evaluation of Spiked Serum Samples

All study data was reviewed and statistically analyzed at NIBSC using the four-parameter logistic model so a consistent approach could be applied. Results from this analysis indicated that candidate sample A and the kit/in-house standards, which in all cases are essentially a dilution of batches of Humira^®^ in appropriate matrix showed comparable dose-response profiles in all laboratories. The suitability of the candidate standard A in measuring levels in a serum matrix in assays in routine use was assessed by expressing levels of adalimumab (μg/ml) quantified in spiked serum samples relative to sample A or either the kit standards (labs 1Ta, 1Tb, 2T, 3T, 4T, 10T, 12T, 14T) or in-house standards (labs 6T, 7T, 8T, 9T, 15T, 16T) as appropriate. For all calculations using candidate A, a concentration of 50 μg per ampoule was assumed. Data from three laboratories was excluded from the main statistical analysis either due to limited data (only one assay) or for non-adherence to study protocol but all data were incorporated when comparing results from laboratories using the same assay.

### Estimates for Adalimumab Levels in Samples Relative to Kit/In-House Standards or Sample A

A summary of combined geometric mean estimates (μg/ml) for samples S1-S24 spiked with adalimumab and a low concentration of anti-adalimumab (ADA) for samples S21-S24, calculated relative to kit/in-house standards and candidate sample A as standard is shown in **Table 9**.

**Table 9:** Summarized estimates for adalimumab content of spiked serum samples.

Sample number	Spiked preparation	Theoretical Level (µg/ml)	Relative to IH/kit			Relative to A		
			Overall GM (µg/ml)	% of expected	Inter-lab GCV (%)	Overall GM (µg/ml)	% of expected	Inter-lab GCV (%)
S2	A (1)	2	2.01	100.5	16.04	2.31	115.5	18.62
S3	A (1)	6	5.83	97.2	13.08	6.24	104.0	12.69
S4	A (1)	12	11.4	95.0	11.70	12.08	100.7	11.97
S6	B (1)	2	1.96	98.0	18.76	2.23	111.5	18.11
S7	B (1)	6	5.82	97.0	16.93	6.24	104.0	16.07
S8	B (1)	12	11.61	96.8	15.34	12.41	103.4	11.04
S10	A (2)	2	1.99	99.5	14.82	2.30	115.0	15.36
S11	A (2)	6	5.98	99.7	12.91	6.42	107.0	11.55
S12	A (2)	12	11.22	93.5	17.75	12.08	100.7	15.28
S14	B (2)	2	1.96	98.0	16.97	2.26	113.0	18.47
S15	B (2)	6	5.81	96.8	15.40	6.23	103.8	14.55
S16	B (2)	12	11.29	94.1	14.57	11.99	99.9	13.91
S17	17/236 (1)	2	2.03	101.5	17.93	2.34	117.0	20.53
S18	17/236 (1)	5	4.84	96.8	13.65	5.28	105.6	15.63
S19	17/236 (2)	2	2.04	102.0	16.06	2.36	118.0	21.15
S20	17/236 (2)	5	4.97	99.4	13.41	5.40	108.0	14.70
S22	A (1)	2 (+ADA)	1.62	81.0	19.27	1.87	93.5	24.05
S23	A (1)	6 (+ADA)	5.48	91.3	16.36	5.92	98.7	15.93
S24	A (1)	12 (+ADA)	11.16	93.0	12.51	11.99	99.9	10.82

IH-In-house standards only; parentheses indicate the serum that was spiked; Serum 1 (First Link), Serum 2 (Sigma).

Samples S1, S5, S9, S13, S21 represent unspiked serum samples except for S21 which was spiked with ADA, however all of these have been omitted from the Table.

**Figure 2:**
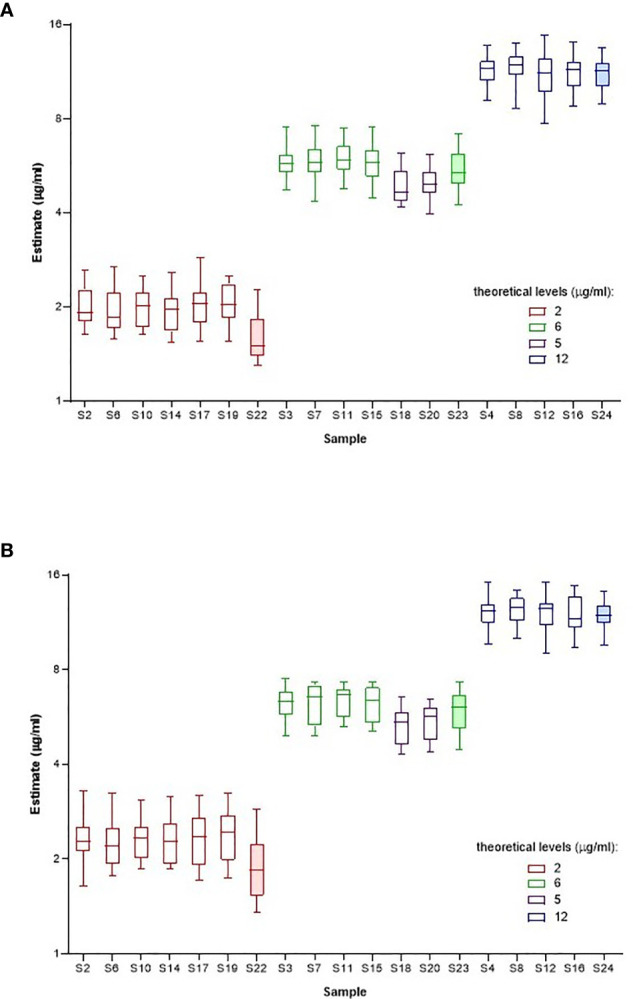
Laboratory geometric mean content estimates (µg/ml) for spiked samples S1-S24 calculated relative to kit or in-house standards **(A)** and Sample A **(B)**. Boxes represent the interquartile range and the line shows the median. The bars represent the range from the maximum to minimum values.

Individual laboratory geometric mean estimates (μg/ml) for samples S1-S24, calculated relative to kit/in-house standards and sample A as standard are summarized in **Supplemental Tables 12** and **13** respectively and illustrated in **Figure 2**. Compared with the theoretical levels, experimentally determined adalimumab levels are systematically higher in a majority of samples (except S22-S24) in most laboratories when calculated relative to A and are lower relative to the kit/in-house standard but this is marginal in both instances. Inter-laboratory variability was comparable regardless of the standard used (median GCV is 15.40% with range 11.70% to 19.27%; median GCV is 15.36% with range 10.82% to 24.05%, **Table 9**) with extreme adalimumab concentrations (2 and 12 μg/ml) showing a large variation. As an example, the estimates for S12 spiked with 12 μg/ml adalimumab ranged from 7.75 to 14.9 μg/ml and 9.06 to 15.2 μg/ml with a GCV of 17.75% and 15.28% relative to kit/in-house standards and sample A respectively.

All study assays were described as detecting only ‘free’ adalimumab. To assess the effect of a low ADA concentration on adalimumab detection, four samples (S21 to S24) containing adalimumab, at 0, 2, 6 or 12 μg/ml were spiked with ADA at 0.5 μg/ml. Although adalimumab levels were slightly reduced in ADA samples compared with similar samples without ADA, the highest impact of ADA was mainly noted at the lowest concentration of adalimumab (2 μg/ml) where the ADA spiked sample showed a decreased adalimumab content relative to counterpart samples devoid of ADA (**Figure 2**).

The study also provided an opportunity, although very limited, to review the results obtained when different laboratories used the same test kit. In the first case, the data obtained by 3 participants (manufacturer and 2 hospital or clinical diagnostic labs) using a commercial ELISA kit points to differences in results obtained by the kit manufacturer and users. Unlike the manufacturer who reported higher levels relative to candidate A, all determined results for the kit users regardless of the standard were lower than the expected theoretical content. However, both users reported similar levels when using candidate A except at the lowest concentrations of adalimumab (2 μg/ml). In the second case, data from 4 participants using a different commercial ELISA (manufacturer and 3 hospital or clinical diagnostic labs) were examined. Similar results for the spiked samples were seen between the manufacturer and a kit user irrespective of the standard used. These results were also consistent with those obtained by other kit users (only 1 assay performed), except at the lower concentration (2 μg/ml). For samples with higher adalimumab amounts, there was a tendency toward better alignment in estimates seen with other laboratories when candidate A was used.

Concordance in log transformed laboratory geometric mean estimates (μg/ml) for the samples calculated relative to kit/in-house standards or sample A as standard is summarized in **Table 10** (values equal to or exceeding 0.90 are shaded). There was generally excellent concordance between laboratories for estimates in spiked serum samples relative to either candidate sample A, the kit standard or in-house reference standards irrespective of the method employed.

**Table 10:** Concordance correlation coefficients for log transformed laboratory geometric mean content estimates (μg/ml) of spiked samples S1-S24 calculated relative to kit or in-house standards **(A)** and Sample A **(B)**.

**A**														
Lab	1Ta	1Tb	2T	3T	4T	6T	7T	8T	9T	10T	12T	14T	15T	16T
1Ta														
1Tb	0.99													
2T	0.99	0.98												
3T	0.97	0.97	0.93											
4T	0.99	0.99	0.99	0.96										
6T	0.96	0.95	0.98	0.89	0.96									
7T	0.99	0.99	0.99	0.96	0.99	0.97								
8T	0.99	0.98	0.99	0.93	0.98	0.97	0.98							
9T	0.98	0.98	0.99	0.92	0.98	0.97	0.98	0.99						
10T	0.96	0.95	0.98	0.87	0.95	0.97	0.96	0.98	0.99					
12T	0.98	0.98	0.95	1.00	0.97	0.91	0.97	0.95	0.94	0.89				
14T	0.94	0.94	0.90	0.99	0.94	0.86	0.93	0.90	0.88	0.82	0.99			
15T	0.99	0.99	0.98	0.98	0.99	0.96	0.99	0.98	0.97	0.93	0.98	0.95		
16T	0.98	0.98	0.99	0.93	0.98	0.97	0.98	0.98	0.99	0.97	0.94	0.88	0.97	
**B**														
Lab	1Ta	1Tb	2T	3T	4T	6T	7T	8T	9T	10T	12T	14T	15T	16T
1Ta														
1Tb	0.99													
2T	0.91	0.88												
3T	0.99	0.99	0.91											
4T	0.96	0.93	0.98	0.96										
6T	0.96	0.95	0.94	0.96	0.97									
7T	0.98	0.99	0.89	0.99	0.95	0.96								
8T	0.97	0.95	0.96	0.97	0.99	0.97	0.96							
9T	0.95	0.92	0.98	0.95	0.99	0.95	0.93	0.99						
10T	0.94	0.91	0.99	0.94	0.99	0.96	0.92	0.98	0.99					
12T	0.95	0.97	0.79	0.96	0.87	0.91	0.97	0.88	0.85	0.84				
14T	0.99	0.99	0.89	0.99	0.95	0.96	0.99	0.96	0.93	0.92	0.98			
15T	0.97	0.96	0.96	0.98	0.99	0.98	0.97	0.99	0.98	0.98	0.90	0.97		
16T	0.97	0.98	0.93	0.99	0.96	0.97	0.98	0.97	0.95	0.94	0.94	0.98	0.98	

Shaded boxes represent > or equal to 90% concordance.

In most assays, determined levels were quite similar to the theoretical content of the samples except for some variation at the extreme adalimumab concentrations. For ELISAs (n=10), values were generally in good agreement with some notable exceptions. For example, a higher value was consistently determined for all samples containing 12 μg/ml in the ELISA in one laboratory (14T) while the ELISA used in another laboratory (10T) indicated a lower value across all concentrations. In both cases, the estimates were improved relative to A. The two ECL assays showed highly consistent and similar estimates for most samples except for those with 12 μg/ml adalimumab. As point-of-care tests, LFI is rapidly gaining momentum - both laboratories reported results that were very similar and close to the theoretical levels of adalimumab in the spiked serum samples for the two assays but discrepancies were observed in samples with 12 μg/ml; one participant consistently reporting very low levels. While one participant showed comparable results regardless of the standard used for calculating adalimumab levels, the other laboratory showed slightly elevated levels relative to candidate A as opposed to the kit standard. Overall, the comparative evaluation of data by assay types (ELISA n=10, ECL n=2, LFI n=2) showed that the geometric mean content obtained in ELISAs for the spiked samples is similar to levels seen with the other assay types except for LFI at the higher concentration of adalimumab (12 μg/ml). To conclude, the utility of Sample A as the common reference standard for the different platforms can only help toward provision of robust and reproducible results and in aligning and harmonizing adalimumab levels across laboratories using the same or different assays.

## Discussion

With a significant number of biosimilar products available for clinical use in EU, the potential for benefits in terms of patient access and reduced costs continues to increase. However, the fierce competition means that the issue of product sustainability is gaining dominance. As a result, manufacturers, whether originator (reference) or biosimilar, are exploring opportunities to drive product selection and commercialization where possible by developing novel approaches (e.g., injector pens, subcutaneous formulation) that provide added value to the patient/prescriber. This is often in parallel with the inevitable post-authorization manufacturing changes that continue for many products, including monoclonal antibodies with modern quality systems and regulatory oversight ensuring that product quality and clinical performance remain unaffected throughout the product’s lifecycle (58–60). Unlike Remicade^®^ (infliximab) and Humira^®^ where a multitude of post-approval changes including site transfers and scale-ups (58–60) did not alter product quality, shifts in quality attributes, particularly in the glycan profiles which influenced functional activity but did not impact clinically were revealed (following extensive interrogation) in batches of a few originator products e.g., Mabthera^®^ (rituximab), Enbrel^®^ (etanercept) (61). However, in the case of Herceptin^®^ (trastuzumab), the downward drift in the proportion of afucosylated glycans and ADCC was thought to be associated with a reduced event-free survival rate in breast cancer patients (three year follow up of a phase 3 study) in comparison with a biosimilar trastuzumab (62, 63). Such examples of drift are extremely rare but with the emergence of biosimilars, concerns regarding product quality both pre- and post-authorization (with potential for divergence from alignment with the originator product at approval as per the biosimilarity paradigm) with impact on clinical performance have resurfaced (64). Consequently, with the current positioning of 8 adalimumab biosimilars in the EU (and of at least 6 in US following product launch in 2023), mitigating measures to minimize this risk and assure consistency in product quality of both originator and biosimilar products are required. The recent establishment (Oct’19) of the adalimumab IS with defined units for individual bioactivities (binding, TNF-α neutralizing, CDC and ADCC) as described here offers a practical solution toward preserving a reliable link between bioassay data and clinical studies throughout the product’s life-cycle subject to its effective utilization as an important tool by key stakeholders (regulators and pharmaceutical industry) for bioassay calibration and validation and for identifying changes in bioactivity and/or controlling drifts where needed.

Results from the multi-center study involving a plethora of assays reflective of the varied mechanism of action of adalimumab in different inflammatory diseases (65) conclusively indicated that both candidate preparations were biologically active, exhibited comparable behavior as illustrated by similarity in dose-response curves in the different functional assays and were suitable for use as reference standards. These findings were not unexpected given both are lyophilizates of approved originator and biosimilar products and have been extensively assessed in comparability studies for regulatory approval. In this study, data analysis was based on setting of equivalence bounds and consistent criteria were applied to assays from all laboratories to assess their relative performance. We found that a large proportion of participant data was of high quality with validity between 75-85% for the different bioassays despite the stringent validity parameters applied for analysis (based on data from coded duplicates) and showed good intermediate precision which resulted in all participant data contributing to the overall potency estimates.

Product testing for potency evaluation requires inclusion of a product-specific reference standard within the bioassay. Therefore, to control product quality in compliance with regulatory guidance, manufacturers develop and establish extensively characterized in-house reference standard(s) for controlling the quality of their specific product for use in a range of applications including potency testing for lot release, for managing changes (e.g. manufacturing processes, tests) and product stability (66). The bioactivity of such “in house” reference standards can vary and their use in deriving relative potency estimates can result in disparate and highly variable potency estimates for a sample when tested in different assays or laboratories. Indeed, a close examination of the bioassay data revealed that when participants’ in-house standards were used, there was a tendency toward discrepant relative potency estimates for the samples in some laboratories reflecting the diversity and differences between in-house standards. This was broadly seen for the multiple activities tested, both Fab- (e.g., binding, neutralization) and Fc-related (e.g., CDC, ADCC) but was most notable for CDC assays which showed the greatest variability in potency (inter-laboratory GCV of 27.56% and 19.11% for samples B and C respectively) across the four laboratories where tested. The low potency largely confined to two laboratories may potentially be related to differences in the critical quality attributes of the in house standards that preferentially influence CDC as opposed to other bioactivities, i.e., differences in Fc glycan pattern, particularly the terminal galactose content, may affect CDC activity (67–69), although an association with particular assay systems cannot be ruled out. Remarkably, ADCC data was quite consistent and associated with a GCV of <19% for samples A, B and C relative to the in-house standards, similar to data from binding assays.

In contrast to the above, a publicly available common reference standard for potency determination can provide consistent and harmonized potency estimates and reduce inter-laboratory variability. This paradigm was also illustrated here as shown (**Table 6**) by the excellent agreement in potency estimates for all the tested activities of adalimumab, when the candidate preparation coded 17/236 was used as a common standard despite differences in assay methodologies across participants. A close agreement in potency estimates for TNF binding and neutralization assays, regardless of the method, was also seen in the case of infliximab when a common standard was used (39). In this study, however, this finding was also extended to other *in vitro* cell-based assays and seems interesting given the complexity of some of these assays. ADCC assays, for example, are highly influenced by the target cell, the effector cell type, the expression of FcγRIIIa receptors, receptor polymorphism, the assay conditions, the readout employed and importantly the glycosylation pattern of the mAb, in particular the degree of afucosylation (50, 52, 62, 69). In this study, three differently engineered target cells (CHO, 3T3 or HEK) expressing membrane bound TNF were used in combination with either engineered Jurkat T cell effectors resulting in a ‘surrogate ADCC assay’ based on effector cell activation or NK cell-lines which promote cellular lysis and provide an end-point killing assay which is considered more physiological and reflects better the mechanism of action of ADCC (70, 71). Interestingly, despite the diversity in the target and effector cells used, the individual potencies in the ADCC assays were quite consistent among laboratories relative to A with values of 0.98 - 0.99 for B (except in one lab with a value of 0.88 and GCV of 18.07%) and 1.04 - 1.07 for C and a GCV of < 25%. Overall, the geometric mean potency estimates from ADCC assays relative to either A or to in-house standards were very close to 1 and very similar to those derived from neutralization assays with low inter-laboratory variability, from 5.44% for B to 9.32% for C, relative to sample A. In fact, sample A reduced the inter-laboratory variability across a range of *in vitro* bioassays and binding assays. For TNF-α neutralization bioassays which employed the 3^rd^ WHO IS for TNF-α (12/154) as the critical reagent (to reduce assay variability) rather than using differently sourced TNF-α, inter-laboratory GCVs of less than 7% relative to A were easily achievable with slightly larger GCVs of less than 10% in all other assays. Furthermore, Sample D, which contained 20% less adalimumab, showed equivalent lower potency estimates in most of the assays where tested. To conclude, there were improvements in potency values and inter-laboratory variability for potency estimates expressed relative to a common standard, sample A in comparison with the in-house standards.

On the basis of the large data set in this study and the stability of sample A on storage (with no degradation at elevated temperatures over 15 months), the suitability of sample A (coded 17/236) to serve as an IS for bioactivity of adalimumab products was confirmed. Therefore, arbitrary independent units of 500 IU, which are not related to any specific method of determination, were assigned for each of the individual bioactivities (binding, TNF-α neutralizing, CDC and ADCC) ascribed to the adalimumab IS (coded 17/236) consistent with other mAb ISs. This approach in consideration of a strategy for a future replacement standard, would allow assignment of independent units for each activity of the replacement standard (when calibrated against the 1^st^ IS to maintain continuity with the IU) in view of the expected variation in the relative ratio of individual bioactivities of different adalimumab products.

From the perspective of adalimumab therapy, the value of routinely measuring trough drug levels for optimizing clinical efficacy is currently being explored (72, 73). Several factors including ADA formation can contribute to sub-therapeutic serum levels and loss of response in some patients (21, 28). Consensus is emerging that while low dosage/concentration of TNF inhibitors may decrease efficacy and increase the risk of ADA, overtreatment should be avoided given the increased risks of side-effects and the significant costs of the medication (31). Therefore, well-defined therapeutic target ranges are needed to guide effective treatment while allowing dose tapering/intensification or a switch to another product within the same product class or another product class with a different mechanism of action, in instances, where a risk to the patient is perceived (28, 31, 73). In several studies, serum adalimumab levels associated with clinical response/efficacy have been proposed (24–26, 29, 74). For example, in adults with RA, adalimumab trough concentrations of 5-8 mcg/ml are thought to be adequate for response to treatment, higher concentrations providing no additional benefit (30, 31, 74). However, optimal cut-off values still need to be established for the different prescribed indications. Accumulating evidence suggests that TDM improves patient outcomes and is cost-effective for inflammatory bowel diseases such as Crohn’s but this is not the case for other indications (29, 72, 73). Poor study design (e.g. retrospective, small size), selection bias (lack of heterogeneity), lack of standardized treatment, ill-defined timing of blood sampling (confounded by ADA) and importantly, the heterogeneity in the assays used for clinical testing have all contributed to inconclusive data (29, 30, 73). Rheumatologists have stipulated requirements for implementing TDM in clinical practice; reliable methods for quantifying therapeutic and ADA, the need for evidence-based guidelines or algorithms to define various therapeutic options (e.g., predicting responsiveness, failure or dose tapering) and finally, the need for patient-specific dosing schedules for adjusting clinical response (73). In the UK, assessment by NICE has concluded that further research needs to be completed on the clinical effectiveness of using TDM ELISA tests for TNF-alpha inhibitors in RA as there is currently insufficient evidence to recommend routine adoption of these tests (75). A similar stance has been adopted by the British Rheumatology association but paradoxically, in Scotland, a national TDM service for adalimumab and infliximab has been introduced. In another development, the European League Against Rheumatism have set up a taskforce to review the evidence on TDM in RA with support from a recently launched clinical trial with the aim of providing recommendations or advice to clinicians (76).

Most commercial kits for quantitation of adalimumab are ELISA-based although point-of-care testing kits (LFI) which are rapid and offer a distinct advantage over other methods have also become available and could be integrated into routine clinical practice (77). However, novel quantitative approaches are also being explored in several laboratories (78, 79). A recently published comparative assessment of the performance of three adalimumab ELISAs and one LFI concluded that the LFI is a reliable alternative to ELISA, and further indicated that some assays systematically measure higher/lower values than others, such differences most likely attributed to variation in ELISA reagents and/or protocols e.g. differences in diluent, in dilution practices and in detection reagents (56). Other publications assessing commercial or in-house adalimumab assays demonstrated good linear correlations between the various assays for recovery and quantitation of adalimumab (50, 80–82). However, the absolute drug concentrations in the analyzed clinical samples (or spiked serum samples) were variable (52, 80–82) and not always interchangeable emphasizing the need to use the same assay to follow patients longitudinally in clinical practice in the absence of a common standard and urging caution when comparing study results from different kits. This conclusion was also drawn in two recent studies comparing commercial assays for measurement of infliximab (all ELISAs) and adalimumab (one LFI, two ELISAs) trough levels. In the infliximab case, despite an excellent correlation of infliximab levels between assays, a substantial variation in some results and systematic biases of infliximab trough levels was noted which could result in divergent therapeutic decisions for some patients (83). Similarly, in the comparative study measuring adalimumab levels in patient sera, a lack of interchangeability between methods was observed, with greater differences noted as ADA levels increased (84). This disagreement in results, evident also in other studies, has led to calls from several groups for the need for standardization of assays for detection of levels of anti-TNFs and ADA in clinical samples (32, 33, 73).

The suitability of the candidate, sample A as reference standard for assays in use for clinical monitoring of adalimumab levels was therefore assessed using some of the above-mentioned methods. Serum samples were spiked with adalimumab preparations (A, B) and levels quantified relative to the assay’s standard or to sample A with the intention of measuring levels in a serum matrix to evaluate assay analytical performance in conditions reflecting the clinical scenario. This also allowed us to assess whether candidate A when used as a common standard would harmonize levels and improve inter-laboratory variability. Overall, the adalimumab content in the spiked serum samples was found to be mostly comparable and consistent with the theoretical content. Some variability in results between laboratories/assays was observed, which was expected as the methodologies used in the study are diverse. However, inter-laboratory assay variability was also comparable regardless of the standard used. Evaluation of correlation coefficients showed excellent inter-laboratory concordance for the spiked samples (equal to or > 0.90 in most laboratories) regardless of the standard or the method employed. Such concordance was also seen when the same ELISA was used in different laboratories e.g., ELISA manufacturer and different users, although some unexpected variability was observed in one of the two instances where the same assay was performed by different users, implicating either batch differences in kit standard and/or analyst-dependent assay discrepancies. All study assays were described as detecting only “free” adalimumab and consistent with this; slightly lower levels of adalimumab were seen in samples spiked with both ADA and adalimumab (at 2μg/ml) relative to their counterpart samples.

Despite the caveat that only a limited number of assay systems were evaluated in the present study, it is evident from the study data that the candidate preparation 17/236 is suitable for use in the tested assay systems and, therefore, can be used for assuring the analytical performance of the different bioanalytical tests available in clinical laboratories and for qualification of in house standards based on the assumed mass content of the ampoule. As TDM relies on accurate quantification of the therapeutic, the use of the 1st WHO IS for adalimumab would allow comparisons of results across different immunoassays/platforms and enable further research, where possible on clinical effectiveness of TDM tests in various indications. Additionally, a common standard will facilitate standardization and harmonization of clinical monitoring assays and, in turn, improve treatment strategies for patients thus fulfilling the demand from clinicians and healthcare organizations (32, 33, 73).

In the long-term, standardization of ADA assays for adalimumab is also anticipated. NIBSC has initiated development of reference antibody/panels for standardizing ADA assays for several therapeutics (e.g., infliximab, adalimumab, rituximab) as part of a WHO program on developing standards for immunogenicity assays for biotherapeutics (85). The 1st reference panel of human antibodies against erythropoietin, established by the WHO is currently available from NIBSC (86). Presently, efforts are underway for standardizing infliximab ADA assays (subject to a successful collaborative study and establishment by WHO of the reference antibody/panel) and will be followed soon after by adalimumab ADA assays. It is anticipated that the reference antibody/panels would help in selection of suitable assays, benchmarking of in-house positive controls/standards where appropriate, facilitate pharmacovigilance and assist in harmonizing and validating ADA detection assays which would be beneficial for TDM practice (87, 88).

**Figure 3:**
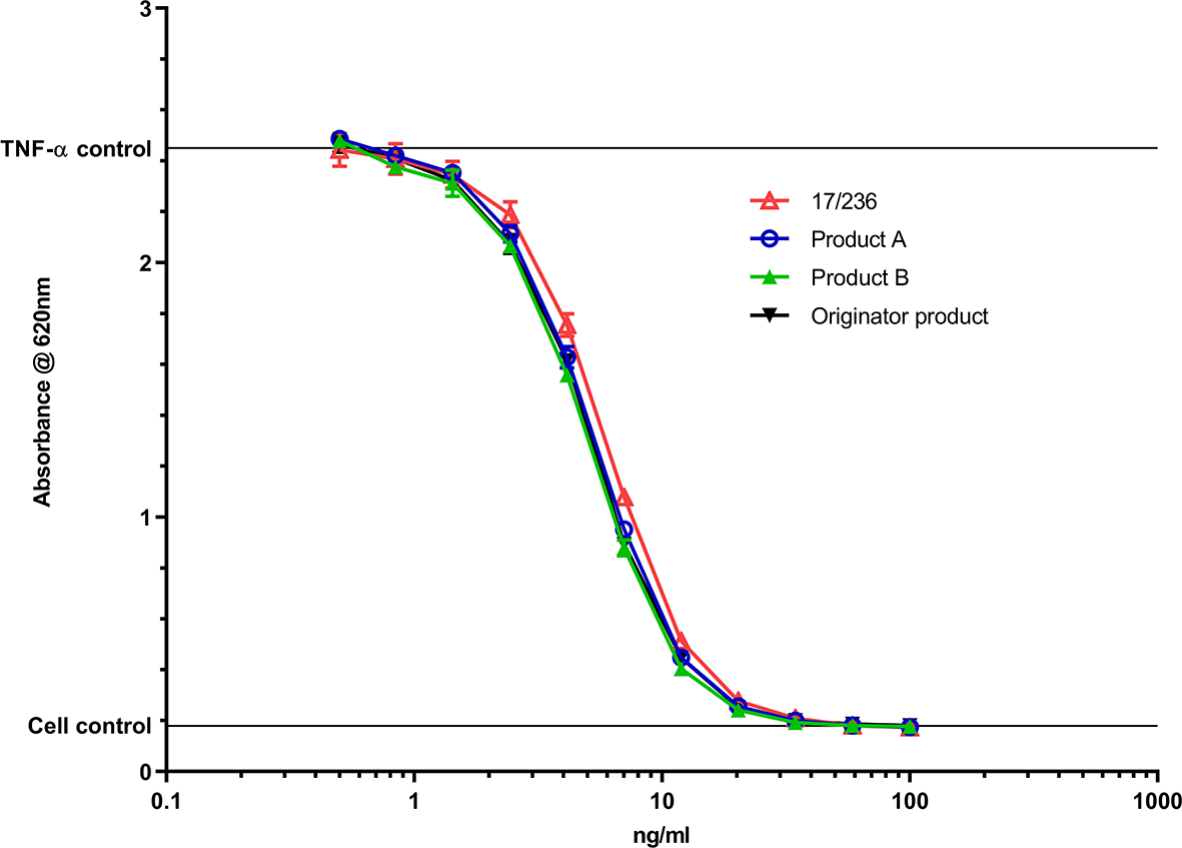
Comparison of TNF-α neutralization activity of the IS 17/236 with 2 biosimilar adalimumab products (a single batch) and the originator product in a HEK Blue CD40L reporter gene assay. Product A – Hulio®; Product B – Hyrimoz®. A fixed concentration of 40IU/ml of TNF-α IS (12/154) was used in the assay.

To summarize, the recent establishment of the WHO IS for adalimumab based on the results of the international collaborative study allows it to be effectively used by stakeholders world-wide in several ways to promote not only product quality but also clinical monitoring in adalimumab treated patients. In its role as a publicly available ‘primary’ standard supporting bioassay performance with 500 IU each for its individual bioactivities (binding, TNF-a neutralizing, CDC and ADCC), the IS will firstly facilitate calibration of secondary standards (manufacturer’s, regional) with traceability to IU and serve as a stability monitoring tool for these local standards. This will help in supporting development of products of consistent quality pre- and post-marketing globally as illustrated in **Figure 3** which shows comparative data of TNF-alpha neutralization activity of the IS 17/236 with marketed adalimumab products; 2 biosimilar products and the originator product in a HEK Blue CD40L reporter gene assay. Secondly, based on its proven ability to harmonize potency values between laboratories, the IS will serve as a ‘benchmark’ for harmonizing bioactivity across products and increase confidence in the rapidly expanding biosimilar market. Thirdly, the IS can be successfully exploited as an important tool in identifying changes in bioactivity and potentially controlling drifts where needed during the life-cycle management of both innovator and biosimilar products. This will assist in harmonizing bioactivity across different products over time and also assure more confidence in the rapidly expanding landscape of biosimilar products. Lastly, the IS can support independent potency testing as required in investigations relating to falsified medicines and post-marketing surveillance activities where necessary. It should be realized that the adalimumab IS with some features in common with other mAb ISs, is a distinct and separate entity from the reference medicinal product (used for biosimilarity determinations) and should not be misused as a reference medicinal product for determining biosimilarity or to define product specific activity or to change current dosing (in mass units) or revise product labeling (36, 38, 39, 89).

From the perspective of clinical monitoring, the mass units of the adalimumab IS will allow calibration of secondary (manufacturer-specific) standards in assays routinely used for quantitating adalimumab in the clinical setting, encourage development of innovative and effective assay systems and assist in assuring analytical assay performance and validation where needed. Importantly, the common standard will facilitate harmonization of clinical assays and assist in formulating treatment algorithms for informed clinical decision-making for better patient outcomes.

To conclude, it is anticipated that the WHO IS will have a significant impact in creating safe and effective adalimumab products of consistent quality, in building more confidence in their prescribing and uptake while enabling progress toward personalized treatment options and effective disease management for realization of full patient benefit.

## Data Availability Statement

The original contributions presented in the study are included in the article/**Supplementary Material**. Further inquiries can be directed to the corresponding author.

## Author Contributions

All authors listed have made a substantial, direct, and intellectual contribution to the work and approved it for publication.

## Funding

This paper is based on independent research commissioned and funded by the National Institute for Health Research (NIHR) Policy Research Program (NIBSC Regulatory Science Research Unit), UK. The views expressed in the publication are those of the author(s) and not necessarily those of the NHS, the NIHR, the Department of Health, ‘arms’ length bodies or other government departments.

## Conflict of Interest

The authors declare that the research was conducted in the absence of any commercial or financial relationships that could be constructed as a potential conflict of interest.
