# Supplementary material for: The First WHO International Standard for Adalimumab: Dual Role in Bioactivity and Therapeutic Drug Monitoring
Source: Front Immunol. 2021 Apr 15;12:636420. doi: 10.3389/fimmu.2021.636420 (PMC8082443; doi:10.3389/fimmu.2021.636420)
Supplement: Supplementary file 1 [file DataSheet_1.docx]

**The first WHO international standard for Adalimumab: Dual role in bioactivity and therapeutic drug monitoring**

**Supplemental Data**

**Supplemental Table 1: Geometric mean potencies of lyophilized trial fill samples relative to the bulk material (n=3) using TNF-α neutralization bioassays**

| Method | Formulation | 95% LCL | GM potency | 95% UCL | GCV % |
| --- | --- | --- | --- | --- | --- |
| L929 | a | 0.87 | 1.03 | 1.23 | 16 |
|  | b | 0.72 | 0.94 | 1.22 | 26 |
| WEHI-164 | a | 0.82 | 0.94 | 1.07 | 12 |
|  | b | 0.7 | 0.91 | 1.19 | 26 |

Adalimumab was formulated and freeze-dried using two formulations: a) 25mM Sodium citrate tribasic dihydrate, 150mM Sodium chloride, 1% (v/v) clinical grade Human serum albumin, HSA, pH 6.5 and b) 10mM L-Histidine, 10mM L-Histidine hydrochloride monohydrate, 1% D-trehalose dihydrate, 0.01% Polysorbate-20, 1% (v/v) clinical grade HSA, pH 6.2.

**Supplemental Table 2: Materials used in final adalimumab candidate preparations^1^**

| **Ampoule code** | **Fill date** | **Study code** | **No of Ampoules in Stock** | **Protein (Predicted Mass - μg)** | **Excipients** |
| --- | --- | --- | --- | --- | --- |
| 17/236^1^ | 25/01/18 | A, C | ~9755^3^ | 50 | 25mM Sodium citrate tribasic dihydrate, 150mM Sodium chloride pH 6.5, 1% Human serum albumin |
| 18/124^1^ | 25/05/18 | B | ~7859 | 50 |  |
| SS711^2^ | 08/08/18 | D | 150 | 40 |  |

^1^ The candidate preparations were expressed in CHO cells; they will be stored at -20^o^C at NIBSC as the custodian laboratory; ^2^ This preparation was produced from the same bulk drug substance as used for 17/236 - this was included for assessing assay sensitivity or ability of the assays to detect differences but is not a candidate standard.^3^All ampoules are intended for use as WHO International standard.

**Supplemental Table 3A: Brief details of TNF-α neutralization assays contributed to the study**

| **Lab**  **code** | **Cell line** | **Assay Type** | **Final TNF-α concentration IU/ml** | **Actinomycin D concentration µg/ml** | **In House standard (IH)** | **Incubation time (hrs)** | **Assay readout** | **Readout reagent** |
| --- | --- | --- | --- | --- | --- | --- | --- | --- |
| 1 | WEHI-164 | Cytotoxicity | 40 | 1 | No | 24 | Absorbance | MTS (CellTiter 96® AQueous One) |
| 2 | WEHI-164 | Cytotoxicity | 60 | 0.5 | No | 23 | Absorbance | WST-8 |
| 3 | WEHI-164 | Cytotoxicity | 80 | 2 | Yes (H) | 24 | Absorbance | MTS/PMS |
| 4a | WEHI-164 | Cytotoxicity | 40 | 2 | Yes (O^£^) | 18-20 | Absorbance | MTT |
| 5a | WEHI-164 | Cytotoxicity | 10 | 1 | No | 20 | Absorbance | MTT |
| 6 | WEHI-164 | Cytotoxicity | 5 | 0.5 | Yes (IH) | 19-24 | Absorbance | CCK-8 |
| 7 | WEHI-13VAR | Cytotoxicity | 15 | 1 | No | 20 | Absorbance | MTS (CellTiter 96® AQueous One) |
| 8a/b | L929 | Cytotoxicity | 20 | 1 | No | 20 - 24 | Absorbance^$^ | Alamar Blue |
| 9 | L929 | Cytotoxicity | 134 | N/A | No | 48 | Absorbance | Crystal violet |
| 10 | L929 | Cytotoxicity | ~12 | 1 | No | 18-20 | Absorbance | MTS |
| 11 | L929 | Cytotoxicity | 20 | 1 | Yes (IH^+^) | 22 | Fluorescence | Resazurin |
| 12 | L929 | Cytotoxicity | 20 | 5 | Yes (IH) | 18-22 | Absorbance | MTS (CellTiter 96® powder) |
| 13 | L929 | Cytotoxicity | 15 | 4 | Yes (IH) | 14-18 | Absorbance | MTS (CellTiter 96® AQueous One) |
| 14 | L929 | Cytotoxicity | 15 | 1 | Yes (IH) | 16-18 | Absorbance | CCK-8 |
| 15 | L929 | Cytotoxicity | 20 | 4 | No | 18-22 | Fluorescence | Alamar blue |
| 16 | L929 | Cytotoxicity | ~ 4 | 20 | Yes (IH) | 16 | Absorbance | CCK-8 |
| 17 | L929 | Cytotoxicity | 5 | 2 | Yes (H) | 20-22 | Absorbance | MTT |
| 18 | L929 | Cytotoxicity | 10 | 1 | No | 20-24 | Luminescence | ATP-Lite |
| 19 | L929 | Cytotoxicity | ~ 134 | N/A | Yes (H) | 48 | Absorbance | Crystal violet |
| 20 | L929 | Cytotoxicity | 10 | 0.1 | Yes (H) | 23-26 | Absorbance | CCK-8 |
| 5b | L929 | Cytotoxicity | 10 | 1 | No | 20 | Absorbance | MTT |
| 4b | HEK293 NF-κB-SEAP | Reporter gene | 40 | N/A | Yes (O^£^) | 22 | Absorbance | Quanti-Blue |
| 8c | HEK293 NF-κB-SEAP | Reporter gene | 40 | N/A | No | 20 - 24 | Absorbance | Quanti-Blue |
| 21 | HEK293 NF-κB-Luc | Reporter gene | 172 | N/A | Yes (H) | 5 | Luminescence | ONE-GloTM Luciferase Assay |
| 22 | HEK293 NF-κB-Luc | Reporter gene | 80 | N/A | Yes (H) | 4 | Luminescence | ONE-GloTM Luciferase Assay |
| 23 | HEK293 NF-κB-Luc | Reporter gene | 50 | N/A | Yes (IH) | 24 | Luminescence | Steady-Glo® Luciferase Assay |
| 24 | HEK293 NF-κB-Luc | Reporter gene | 100 | N/A | Yes (H) | 5 | Luminescence | DualGlo Luciferase assay |
| 26a | HEK293 NF-κB-Luc | Reporter gene | 172 | N/A | Yes (IH) | 16-24 | Luminescence | Steady Glo Luciferase assay |
| 2 | U937 | Apoptosis | 40 | N/A | No | 2.5 | Luminescence | Caspase-Glo 3/7 |
| 25 | U937 | Apoptosis | 2000 | N/A | Yes (IH) | 3.5 | Luminescence | Caspase-Glo 3/7 |
| 26b | U937 | Apoptosis | 172 | N/A | Yes (IH) | 4 | Luminescence | Caspase-Glo 3/7 |

H : Humira®; IH : in-house/proprietary therapeutic adalimumab; IH^+^ : in-house research grade adalimumab; O^£^: another TNF antagonist (non adalimumab) ; ^$^both absorbance and fluorescence measured

**Supplemental Table 3B: Brief details of ADCC, CDC and cell binding assays contributed to the study**

| **Lab**  **Code** | **Bioactivity** | **Source of complement** | **Effector cells (E)** | **Target cells (T)** | **Ratio E:T** | **Assay Type** | **In house standard (IH)** | **Assay duration (hrs)** | **Assay readout** | **Readout reagent** |
| --- | --- | --- | --- | --- | --- | --- | --- | --- | --- | --- |
| 12 | ADCC | N/A | Jurkat-NFAT-luc-FcγRllla | CHO-mTNFα | 1:10 | Reporter gene | Yes (IH) | 4 – 6 | Luminescence | Bright-Glo |
| 16 | ADCC | N/A | NK92- Fcγ­Rllla | CHO-mTNFα | 5:1 | NK cell killing | Yes (IH) | 4 | Absorbance | Cytotoxicity detection kit PLUS (LDH) |
| 23 | ADCC | N/A | NK92- Fcγ­Rllla | 3T3-mTNFα | 1:1 | NK cell killing | Yes (IH) | 4 | Luminescence | CytoTox-Glo |
| 25 | ADCC | N/A | Jurkat-NFAT-luc-FcγRllla | CHO-mTNFα | 1:1 | Reporter gene | No | 20 | Luminescence | Bio-Glo |
| 26 | ADCC | N/A | NK 3.3 | HEK-mTNFα | 10:1 | NK cell killing | Yes (IH) | 1 | Fluorescence | Calcein-AM |
| 6 | CDC | human | N/A | Jurkat-mTNFα | N/A | Viability | Yes (IH) | 2 | Absorbance | CCK-8 |
| 16 | CDC | rabbit | N/A | CHO-mTNFα | N/A | Viability | Yes (IH) | 4 | Luminescence | CellTiter-Glo |
| 25 | CDC | human | N/A | CHO-mTNFα | N/A | Viability | Yes (IH) | 4 | Luminescence | CellTiter-Glo |
| 26 | CDC | human | N/A | Jurkat-mTNFα | N/A | Viability | Yes (IH) | 2 | Luminescence | CellTiter-Glo |
| 12b | Cell binding | N/A | N/A | CHO-mTNFα | N/A | Flow cytometry | Yes (IH) | 1 – 1.5 | Fluorescence | Anti-human IgG (H+L)-FITC |
| 25b | Cell binding | N/A | N/A | CHO-mTNFα | N/A | Flow cytometry | Yes (IH) | 1 | Fluorescence | Anti-Human IgG Fc-PE |

IH : in-house/proprietary therapeutic adalimumab used in all cases.

**Supplemental Table 3C: Brief details of binding assays contributed to the study**

| **Lab code** | **Assay Type** | **In house standard (IH)** | **Assay description** | **Detection reagent** | **Assay readout** | **Readout reagent** |
| --- | --- | --- | --- | --- | --- | --- |
| 7 | ELISA kit | Yes (IH^1^) | Adalimumab binds to TNF-α coated plates. | Anti-human IgG Fc-HRP | Absorbance | TMB |
| 8 | Bridging  ECL | Yes (H) | Adalimumab binds to Biotinylated and Sulfo-Tag labelled TNF-α, captured onto streptavidin coated plates. | Biotinylated + Sulfo Tag TNF-α | Electrochemiluminescence | MSD Read buffer |
| 10 | ELISA | No | Adalimumab binds to TNF-α coated plates. | Anti-human IgG-HRP | Absorbance | TMB |
| 12a | ELISA | Yes (IH) | Adalimumab binds to TNF-α coated plates. | Anti-human Kappa-HRP | Absorbance | TMB |
| 20 | ELISA | Yes (H) | Adalimumab binds to TNF-α coated plates. | Anti-human IgG-HRP | Absorbance | TMB |
| 23 | TR-FRET | Yes (IH) | Europium labelled adalimumab and Cy5 labelled TNF-α form fluorescent complex which is competitively inhibited by unlabelled adalimumab | Europium labelled adalimumab + Cy5 labelled TNF-α | Fluorescence | N/A |
| 25a | ELISA | Yes (IH) | Adalimumab binds to TNF-α coated plates. | Anti-human IgG Fc-HRP | Absorbance | TMB |
| 3 | Biolayer Interferometry | Yes (H) | Adalimumab binds to biotinylated TNF-α captured onto streptavidin biosensor. | N/A | Response binding rate (nm/s) | N/A |
| 12 | SPR | Yes (IH) | Adalimumab captured onto sensor chip immobilised with Anti Human IgG Fc, followed by concentrations of TNF-α | N/A | Response units: expressed as Equilibrium affinity constant KD (M) | N/A |

H = Humira; IH = proprietary adalimumab; IH^1^ – kit standard

**Supplemental Table 4: Percentage of invalid assays per laboratory (Bioassay Study)**

| Assay | Lab | % of assays invalid *vs*  sample A | | | % of assays invalid vs  in-house reference | | | |
| --- | --- | --- | --- | --- | --- | --- | --- | --- |
|  |  | Sample B | Sample C | Sample D | Sample A | Sample B | Sample C | Sample D |
| Neut | 01 | 0 | 0 | - | - | - | - | - |
| Neut | 02a | 0 | 0 | - | - | - | - | - |
| Neut | 02b | 22.2 | 11.1 | - | - | - | - | - |
| Neut | 03 | 40 | 15 | - | 50 | 50 | 40 | - |
| Neut | 04a | 0 | 0 | 22.2 | 100 | 100 | 100 | 100 |
| Neut | 04b | 0 | 0 | 0 | 91.7 | 100 | 88.9 | 100 |
| Neut | 05a | 33.3 | 46.7 | - | - | - | - | - |
| Neut | 05b | 26.7 | 33.3 | - | - | - | - | - |
| Neut | 06 | 0 | 0 | 0 | 0 | 0 | 0 | 0 |
| Neut | 07 | 0 | 0 | - | - | - | - | - |
| Neut | 08a | 0 | 0 | 0 | - | - | - | - |
| Neut | 08b | 0 | 0 | 0 | - | - | - | - |
| Neut | 08c | 0 | 0 | 0 | - | - | - | - |
| Neut | 09 | 33.3 | 8.3 | - | - | - | - | - |
| Neut | 10 | 0 | 0 | 0 | - | - | - | - |
| Neut | 11 | 0 | 11.1 | - | 0 | 0 | 0 | - |
| Neut | 12 | 0 | 0 | - | 0 | 0 | 0 | - |
| Neut | 13 | 0 | 0 | - | 0 | 0 | 0 | - |
| Neut | 14 | 0 | 0 | - | 0 | 0 | 0 | - |
| Neut | 15 | 0 | 0 | - | - | - | - | - |
| Neut | 16 | 0 | 0 | - | 0 | 0 | 0 | - |
| Neut | 17 | 0 | 0 | - | 0 | 0 | 22.2 | - |
| Neut | 18 | 0 | 0 | 0 | - | - | - | - |
| Neut | 19 | 11.1 | 11.1 | 33.3 | 16.7 | 11.1 | 11.1 | 33.3 |
| Neut | 20 | 0 | 0 | - | 0 | - | 0 | - |
| Neut | 21 | 22.2 | 22.2 | - | 0 | 11.1 | 11.1 | - |
| Neut | 22 | 11.1 | 22.2 | - | 0 | 11.1 | 11.1 | - |
| Neut | 23 | 0 | 0 | 0 | 0 | 0 | 0 | 0 |
| Neut | 24 | 33.3 | 33.3 | 33.3 | 66.7 | 66.7 | 11.1 | 33.3 |
| Neut | 25 | 33.3 | 33.3 | - | 0 | 0 | 0 | - |
| Neut | 26a | 0 | 0 | - | 0 | 0 | 0 | - |
| Neut | 26b | 0 | 0 | - | 25 | 25 | 50 | - |
| ADCC | 12 | 0 | 0 | - | 0 | 11.1 | 11.1 | - |
| ADCC | 16 | 0 | 44.4 | - | 11.1 | 0 | 11.1 | - |
| ADCC | 23 | 0 | 0 | 0 | 0 | 33.3 | 0 | 0 |
| ADCC | 25 | 0 | 33.3 | - | - | - | - | - |
| ADCC | 26 | 0 | 33.3 | - | 0 | 0 | 33.3 | - |
| Binding | 03 | 0 | 66.7 | - | 66.7 | 33.3 | 66.7 | - |
| Binding | 07 | 0 | 0 | - | 0 | 50 | 0 | - |
| Binding | 08 | 0 | 0 | 0 | 0 | 0 | 0 | 0 |
| Binding | 10 | 0 | 0 | 0 | - | - | - | - |
| Binding | 12a | 11.1 | 0 | - | 0 | 0 | 0 | - |
| Binding | 12b | 0 | 11.1 | - | 0 | 0 | 22.2 | - |
| Binding | 20 | 0 | 0 | 0 | 5.6 | 0 | 0 | 11.1 |
| Binding | 23 | 0 | 0 | 0 | 16.7 | 0 | 0 | 33.3 |
| Binding | 25a | 0 | 33.3 | - | 0 | 0 | 33.3 | - |
| Binding | 25b | 0 | 0 | - | 0 | 33.3 | 0 | - |
| CDC | 06 | 0 | 0 | 0 | 0 | 0 | 0 | 33.3 |
| CDC | 16 | 11.1 | 33.3 | - | 22.2 | 0 | 0 | - |
| CDC | 25 | 0 | 0 | - | 66.7 | 33.3 | 66.7 | - |
| CDC | 26 | 0 | 0 | - | 0 | 0 | 0 | - |

- Denotes no data

**Supplemental Table 5: Laboratory geometric mean relative potency estimates for neutralization assays**

| **Cell line** | **Lab** | **Potencies relative to sample A** | | | | | | | | | **Potencies relative to in-house reference** | | | | | | | | | | | |
| --- | --- | --- | --- | --- | --- | --- | --- | --- | --- | --- | --- | --- | --- | --- | --- | --- | --- | --- | --- | --- | --- | --- |
|  |  | **Sample B** | | | **Sample C** | | | **Sample D** | | | **Sample A** | | | **Sample B** | | | **Sample C** | | | **Sample D** | | |
|  |  | **GM** | **GCV** | **N** | **GM** | **GCV** | **N** | **GM** | **GCV** | **N** | **GM** | **GCV** | **N** | **GM** | **GCV** | **N** | **GM** | **GCV** | **N** | **GM** | **GCV** | **N** |
| WEHI-164 | 01 | 1.04 | 6.16 | 9 | 1.00 | 6.08 | 9 | - | - | - | - | - | - | - | - | - | - | - | - | - | - | - |
| WEHI-164 | 02a | 1.00 | 9.51 | 9 | 0.99 | 8.28 | 9 | - | - | - | - | - | - | - | - | - | - | - | - | - | - | - |
| WEHI-164 | 03 | 0.92 | 7.84 | 12 | 0.94 | 14.47 | 17 | - | - | - | 0.97 | 17.73 | 10 | 0.94 | 15.31 | 10 | 0.90 | 19.28 | 12 | - | - | - |
| WEHI-164 | 04a^1,2^ | 1.04 | 10.61 | 9 | 0.97 | 12.40 | 9 | 0.82 | 5.77 | 7 | - | - | - | - | - | - | - | - | - | - | - | - |
| WEHI-164 | 05a | 1.24 | 26.59 | 10 | 0.88 | 25.54 | 8 | - | - | - | - | - | - | - | - | - | - | - | - | - | - | - |
| WEHI-164 | 06 | 0.96 | 821 | 6 | 1.05 | 8.71 | 6 | 0.94 | 8.49 | 3 | 0.99 | 8.08 | 15 | 1.01 | 7.69 | 12 | 0.99 | 8.40 | 12 | 0.92 | 7.51 | 3 |
| WEHI-13VAR | 07 | 1.06 | 4.35 | 9 | 1.02 | 6.91 | 9 | - | - | - | - | - | - | - | - | - | - | - | - | - | - | - |
| L929 | 05b | 1.03 | 25.38 | 11 | 1.11 | 20.76 | 10 | - | - | - | - | - | - | - | - | - | - | - | - | - | - | - |
| L929 | 08a | 1.07 | 5.62 | 9 | 1.05 | 9.17 | 9 | 0.85 | 7.11 | 9 | - | - | - | - | - | - | - | - | - | - | - | - |
| L929 | 08b | 1.08 | 7.09 | 9 | 1.04 | 10.23 | 9 | 0.86 | 7.31 | 9 | - | - | - | - | - | - | - | - | - | - | - | - |
| L929 | 09 | 0.97 | 5.83 | 8 | 0.97 | 7.75 | 11 | - | - | - | - | - | - | - | - | - | - | - | - | - | - | - |
| L929 | 10 | 1.02 | 6.44 | 9 | 1.01 | 8.27 | 9 | 0.81 | 15.41 | 3 | - | - | - | - | - | - | - | - | - | - | - | - |
| L929 | 11 | 1.04 | 19.69 | 9 | 1.02 | 22.61 | 8 | - | - | - | 0.71 | 20.89 | 6 | 0.74 | 17.02 | 6 | 0.71 | 20.39 | 6 | - | - | - |
| L929 | 12 | 1.04 | 5.86 | 9 | 0.99 | 7.72 | 9 | - | - | - | 0.89 | 6.10 | 9 | 0.93 | 7.00 | 9 | 0.89 | 5.60 | 9 | - | - | - |
| L929 | 13 | 1.01 | 10.11 | 3 | 1.06 | 14.99 | 3 | - | - | - | 0.99 | 14.59 | 3 | 1.00 | 4.20 | 3 | 1.05 | 5.39 | 3 | - | - | - |
| L929 | 14 | 1.01 | 7.46 | 9 | 0.98 | 7.78 | 9 | - | - | - | 0.91 | 10.16 | 9 | 0.92 | 10.01 | 9 | 0.89 | 4.85 | 9 | - | - | - |
| L929 | 15 | 1.13 | 6.54 | 9 | 1.01 | 7.81 | 9 | - | - | - | - | - | - | - | - | - | - | - | - | - | - | - |
| L929 | 16 | 0.97 | 7.10 | 9 | 0.97 | 5.82 | 9 | - | - | - | 1.01 | 5.46 | 9 | 0.98 | 8.14 | 9 | 0.98 | 5.09 | 9 | - | - | - |
| L929 | 17 | 1.09 | 16.60 | 9 | 1.10 | 13.25 | 9 | - | - | - | 0.95 | 19.68 | 9 | 1.04 | 20.60 | 9 | 0.98 | 14.00 | 7 | - | - | - |
| L929 | 18 | 1.00 | 2.74 | 9 | 0.97 | 2.73 | 9 | 0.80 | 3.27 | 9 | - | - | - | - | - | - | - | - | - | - | - | - |
| L929 | 19 | 1.05 | 4.03 | 8 | 1.00 | 2.47 | 8 | 0.81 | n/a | 2 | 1.01 | 4.90 | 10 | 1.06 | 3.48 | 8 | 1.01 | 3.52 | 8 | 0.81 | n/a | 2 |
| L929 | 20 | 1.06 | 8.32 | 9 | 0.96 | 18.06 | 12 | - | - | - | 1.19 | 2.23 | 3 | - | - | - | 1.10 | 2.55 | 3 | - | - | - |
| NF-κB-SEAP | 04b^1^ | 1.03 | 2.76 | 9 | 1.00 | 3.18 | 9 | 0.85 | 3.75 | 3 | - | - | - | - | - | - | - | - | - | - | - | - |
| NF-κB-SEAP | 08c | 1.02 | 4.65 | 15 | 0.98 | 4.88 | 12 | 0.81 | 2.80 | 12 | - | - | - | - | - | - | - | - | - | - | - | - |
| NF-κB-Luc | 21 | 1.06 | 4.05 | 7 | 1.03 | 4.84 | 7 | - | - | - | 1.15 | 6.00 | 9 | 1.20 | 2.78 | 8 | 1.16 | 3.44 | 8 | - | - | - |
| NF-κB-Luc | 22 | 0.96 | 11.25 | 8 | 1.04 | 11.22 | 7 | - | - | - | 0.97 | 11.64 | 9 | 1.02 | 12.15 | 8 | 1.06 | 15.36 | 8 | - | - | - |
| NF-κB-Luc | 23 | 1.05 | 3.86 | 3 | 1.02 | 2.27 | 3 | 0.81 | 11.92 | 3 | 0.93 | 2.00 | 6 | 0.98 | 3.58 | 3 | 0.95 | 1.50 | 3 | 0.76 | 9.42 | 3 |
| NF-κB-Luc | 24 | 1.18 | 26.35 | 6 | 1.17 | 32.02 | 6 | 1.07 | n/a | 2 | 0.80 | 38.12 | 4 | 1.59 | 52.03 | 3 | 0.84 | 81.03 | 8 | 0.50 | n/a | 2 |
| NF-κB-Luc | 26a | 1.00 | 2.90 | 3 | 1.03 | 7.81 | 3 | - | - | - | 0.96 | 3.85 | 3 | 0.96 | 4.92 | 3 | 0.98 | 9.19 | 3 | - | - | - |
| U937 | 02b | 1.13 | 13.73 | 7 | 1.01 | 14.11 | 8 | - | - | - | - | - | - | - | - | - | - | - | - | - | - | - |
| U937 | 25 | 0.99 | n/a | 2 | 1.02 | n/a | 2 | - | - | - | 0.92 | 7.18 | 3 | 0.92 | 13.35 | 3 | 0.93 | 6.31 | 3 | - | - | - |
| U937 | 26b | 0.96 | 29.98 | 4 | 0.91 | 19.84 | 4 | - | - | - | 1.21 | 35.54 | 3 | 1.16 | 69.92 | 3 | 1.22 | n/a | 2 | - | - | - |

GM: Geometric Mean; GCV: Geometric Coefficient of Variation (%); N: Number of estimates used in calculation of GM and GCV; ^1^ : potencies relative to IH standard excluded as a different TNF antagonist used as IH standard; ^2^  : potencies calculated using a parallel line model

**Supplemental Table 6: Laboratory geometric mean relative potency estimates for ADCC and CDC assays**

| **Assay** | **Lab** | **Potencies relative to sample A** | | | | | | | | | **Potencies relative to in-house reference** | | | | | | | | | | | |
| --- | --- | --- | --- | --- | --- | --- | --- | --- | --- | --- | --- | --- | --- | --- | --- | --- | --- | --- | --- | --- | --- | --- |
|  |  | **Sample B** | | | **Sample C** | | | **Sample D** | | | **Sample A** | | | **Sample B** | | | **Sample C** | | | **Sample D** | | |
|  |  | **GM** | **GCV** | **N** | **GM** | **GCV** | **N** | **GM** | **GCV** | **N** | **GM** | **GCV** | **N** | **GM** | **GCV** | **N** | **GM** | **GCV** | **N** | **GM** | **GCV** | **N** |
| ADCC | 12 | 0.99 | 22.85 | 9 | 1.04 | 18.75 | 9 | - | - | - | 0.93 | 24.48 | 9 | 0.94 | 23.10 | 8 | 0.93 | 22.27 | 8 | - | - | - |
| ADCC | 16 | 0.98 | 16.78 | 9 | 1.07 | 20.81 | 5 | - | - | - | 0.86 | 19.00 | 8 | 0.84 | 22.41 | 9 | 0.90 | 18.26 | 8 | - | - | - |
| ADCC | 23 | 0.99 | 6.91 | 3 | 1.06 | 15.10 | 3 | 0.79 | 6.33 | 3 | 1.07 | 19.69 | 6 | 1.05 | n/a | 2 | 1.05 | 7.73 | 3 | 0.91 | 9.61 | 3 |
| ADCC | 25 | 0.99 | 23.84 | 3 | 1.06 | n/a | 2 | - | - | - | - | - | - | - | - | - | - | - | - | - | - | - |
| ADCC | 26 | 0.88 | 18.07 | 3 | 0.87 | n/a | 2 | - | - | - | 1.26 | 13.48 | 3 | 1.10 | 33.86 | 3 | 1.12 | n/a | 2 | - | - | - |
| CDC | 06 | 1.01 | 11.46 | 9 | 1.11 | 9.81 | 9 | 0.73 | 8.07 | 3 | 0.92 | 12.71 | 12 | 0.90 | 13.46 | 9 | 0.99 | 7.79 | 9 | 0.69 | n/a | 2 |
| CDC | 16 | 1.14 | 27.92 | 8 | 1.08 | 36.07 | 6 | - | - | - | 0.97 | 14.73 | 7 | 1.12 | 16.88 | 9 | 1.01 | 17.35 | 9 | - | - | - |
| CDC | 25 | 1.03 | 9.06 | 3 | 1.13 | 16.27 | 3 | - | - | - | 0.66 | n/a | 1 | 0.67 | n/a | 2 | 0.79 | n/a | 1 | - | - | - |
| CDC | 26 | 0.90 | 2.71 | 3 | 0.93 | 5.30 | 3 | - | - | - | 0.76 | 7.61 | 3 | 0.68 | 10.05 | 3 | 0.71 | 10.10 | 3 | - | - | - |

GM: Geometric Mean; GCV: Geometric Coefficient of Variation (%); N: Number of estimates used in calculation of GM and GCV

**Supplemental Table 7: Laboratory geometric mean relative potency estimates for binding assays**

| **Assay** | **Lab** | **Potencies relative to sample A** | | | | | | | | | **Potencies relative to in-house reference** | | | | | | | | | | | |
| --- | --- | --- | --- | --- | --- | --- | --- | --- | --- | --- | --- | --- | --- | --- | --- | --- | --- | --- | --- | --- | --- | --- |
|  |  | **Sample B** | | | **Sample C** | | | **Sample D** | | | **Sample A** | | | **Sample B** | | | **Sample C** | | | **Sample D** | | |
|  |  | **GM** | **GCV** | **N** | **GM** | **GCV** | **N** | **GM** | **GCV** | **N** | **GM** | **GCV** | **N** | **GM** | **GCV** | **N** | **GM** | **GCV** | **N** | **GM** | **GCV** | **N** |
| Binding | 03 | 1.00 | 3.23 | 3 | 1.02 | n/a | 1 | - | - | - | 1.04 | n/a | 1 | 1.01 | n/a | 2 | 1.02 | n/a | 1 | - | - | - |
| Binding | 07^1^ | 0.89 | n/a | 2 | 0.96 | n/a | 2 | - | - | - | 0.67 | n/a | 2 | 0.70 | n/a | 1 | 0.64 | n/a | 2 | - | - | - |
| Binding | 08^1^ | 0.98 | 6.78 | 9 | 0.98 | 9.00 | 9 | 0.80 | 9.09 | 9 | 0.93 | 7.00 | 9 | 0.91 | 8.76 | 9 | 0.91 | 9.85 | 9 | 0.74 | 8.38 | 9 |
| Binding | 10 | 0.99 | 5.67 | 9 | 0.96 | 7.11 | 9 | 0.80 | 6.18 | 9 | - | - | - | - | - | - | - | - | - | - | - | - |
| Binding | 12a | 1.16 | 11.54 | 8 | 1.01 | 13.62 | 9 | - | - | - | 0.94 | 15.26 | 9 | 1.05 | 15.83 | 9 | 0.96 | 11.44 | 9 | - | - | - |
| Binding | 20 | 1.04 | 16.47 | 9 | 1.09 | 13.06 | 9 | 0.77 | 12.96 | 9 | 0.90 | 19.78 | 17 | 0.91 | 23.21 | 9 | 0.96 | 15.14 | 9 | 0.73 | 26.42 | 8 |
| Binding | 23 | 0.98 | 24.01 | 3 | 1.02 | 0.61 | 3 | 0.94 | 11.18 | 3 | 0.90 | 19.41 | 5 | 0.94 | 18.45 | 3 | 0.98 | 16.99 | 3 | 0.77 | n/a | 2 |
| Binding | 25a | 0.99 | 32.32 | 3 | 1.19 | n/a | 2 | - | - | - | 0.77 | 28.08 | 3 | 0.76 | 14.32 | 3 | 0.92 | n/a | 2 | - | - | - |
| Binding (cell based) | 12b | 1.06 | 8.72 | 9 | 1.03 | 10.57 | 8 | - | - | - | 0.93 | 10.83 | 9 | 0.98 | 16.36 | 9 | 0.99 | 9.80 | 7 | - | - | - |
| Binding (cell based) | 25b | 1.09 | 9.98 | 3 | 0.99 | 15.60 | 3 | - | - | - | 1.05 | 16.16 | 3 | 1.12 | n/a | 2 | 1.04 | 4.48 | 3 | - | - | - |

GM: Geometric Mean; GCV: Geometric Coefficient of Variation (%); N: Number of estimates used in calculation of GM and GCV; ^1^ : potencies calculated using a parallel line model

**Supplemental Table 8: Laboratory geometric mean ED50 estimates (ng) and final TNF-α concentrations for neutralization assays**

| Cell line | Lab | Final TNF-α concentration in assay IU/ml | GM | | | | | GCV | | | | |
| --- | --- | --- | --- | --- | --- | --- | --- | --- | --- | --- | --- | --- |
|  |  |  | A | B | C | D | IH | A | B | C | D | IH |
| WEHI-164 | 01 | 40 | 12.31 | 11.84 | 12.28 | - | - | 18.98 | 16.72 | 21.44 | - | - |
| WEHI-164 | 02a | 60 | 32.54 | 32.57 | 32.97 | - | - | 11.74 | 11.74 | 11.46 | - | - |
| WEHI-164 | 03 | 80 | 11.00 | 13.14 | 13.21 | - | 10.48 | 74.85 | 51.47 | 56.43 | - | 76.83 |
| WEHI-164 | 04a^1^ | 40 | - | - | - | - | - | - | - | - | - | - |
| WEHI-164 | 05a | 10 | 17.06 | 15.25 | 17.60 | - | - | 32.05 | 27.84 | 34.10 | - | - |
| WEHI-164 | 06 | 5 | 1.70 | 1.67 | 1.77 | 1.88 | 1.71 | 33.54 | 37.34 | 35.39 | 3.66 | 32.74 |
| WEHI-13VAR | 07 | 15 | 19.30 | 18.28 | 18.87 | - | - | 24.35 | 22.14 | 17.19 | - | - |
| L929 | 05b | 10 | 12.74 | 11.96 | 11.63 | - | - | 64.38 | 71.41 | 60.54 | - | - |
| L929 | 08a | 20 | 8.53 | 7.94 | 8.13 | 9.98 | - | 4.92 | 6.93 | 6.96 | 5.44 | - |
| L929 | 08b | 20 | 8.04 | 7.45 | 7.71 | 9.37 | - | 4.77 | 7.20 | 7.37 | 4.27 | - |
| L929 | 09 | 134 | 7.36 | 7.55 | 7.48 | - | - | 14.70 | 13.98 | 13.57 | - | - |
| L929 | 10 | 11.625 | 15.99 | 15.15 | 15.41 | 21.57 | - | 13.90 | 14.94 | 15.78 | 20.85 | - |
| L929 | 11 | 20 | 5.39 | 5.20 | 5.19 | - | 3.49 | 31.35 | 30.47 | 32.76 | - | 17.24 |
| L929 | 12 | 20 | 11.30 | 10.83 | 11.37 | - | 10.09 | 6.00 | 6.22 | 6.33 | - | 7.90 |
| L929 | 13 | 15 | 5.03 | 4.98 | 4.73 | - | 4.99 | 7.85 | 4.49 | 10.43 | - | 7.39 |
| L929 | 14 | 15 | 4.62 | 4.59 | 4.73 | - | 4.21 | 12.94 | 13.50 | 8.80 | - | 8.71 |
| L929 | 15 | 20 | 10.43 | 9.25 | 10.34 | - | - | 20.74 | 15.64 | 28.40 | - | - |
| L929 | 16 | 3.58 | 46.01 | 47.42 | 47.36 | - | 46.55 | 8.31 | 7.86 | 7.35 | - | 4.29 |
| L929 | 17 | 5 | 6.85 | 6.28 | 6.20 | - | 6.51 | 19.16 | 5.87 | 27.84 | - | 19.54 |
| L929 | 18 | 10 | 10.13 | 10.16 | 10.42 | 12.64 | - | 8.58 | 9.92 | 9.64 | 9.10 | - |
| L929 | 19 | 134.4 | 8.85 | 8.45 | 8.83 | 10.91 | 8.90 | 1.94 | 4.51 | 3.01 | n/a | 4.86 |
| L929 | 20 | 10 | 5.40 | 5.26 | 5.60 | - | 5.89 | 16.84 | 22.43 | 17.01 | - | 10.00 |
| HEK293 NF-κB-SEAP | 04b | 40 | 16.23 | 15.69 | 16.24 | 19.25 | - | 7.00 | 8.68 | 6.86 | 3.00 | - |
| HEK293 NF-κB-SEAP | 08c | 40 | 11.36 | 11.25 | 11.56 | 14.04 | - | 10.02 | 9.74 | 11.00 | 10.55 | - |
|  |  |  |  |  |  |  |  |  |  |  |  |  |
| HEK293 NF-κB-Luc | 21 | 172 | 36.22 | 35.27 | 35.53 | - | 41.74 | 10.35 | 10.09 | 9.25 | - | 7.02 |
| HEK293 NF-κB-Luc | 22 | 80 | 45.68 | 45.27 | 41.83 | - | 44.40 | 37.08 | 22.68 | 23.90 | - | 30.35 |
| HEK293 NF-κB-Luc | 23 | 50 | 8.52 | 7.98 | 8.25 | 10.64 | 7.95 | 9.00 | 13.19 | 12.37 | 9.25 | 7.85 |
| HEK293 NF-κB-Luc | 24 | 100 | 58.89 | 46.61 | 49.29 | 58.64 | 36.88 | 9.97 | 31.68 | 32.56 | 6.40 | 38.78 |
| HEK293 NF-κB-Luc | 26a | 172 | 20.60 | 20.51 | 20.07 | - | 19.73 | 5.22 | 2.28 | 2.46 | - | 6.92 |
| U937 | 02b | 40 | 12.79 | 11.69 | 11.97 | - | - | 34.99 | 36.45 | 32.44 | - | - |
| U937 | 25 | 2000 | 178.61 | 178.24 | 175.03 | - | 163.47 | 12.63 | 19.10 | 11.73 | - | 5.11 |
| U937 | 26b | 172 | 187.30 | 195.17 | 205.19 | - | 218.67 | 33.96 | 9.38 | 32.90 | - | 62.49 |

GM: Geometric Mean; GCV: Geometric Coefficient of Variation (%); IH: In-house reference; ^1^ : ED50 values not calculated as parallel line model used for analysis (see statistical analysis section)

**Supplemental Table 9: Summary of results from reconstitution stability studies of candidate preparation 17/236 assayed using an L929 cell cytotoxicity assay**

| Temperature (°C) | Time (Days) | LCL | Relative Potency to a freshly reconstituted ampoule | UCL |
| --- | --- | --- | --- | --- |
| +4 | 1 | 0.90 | 0.98 | 1.07 |
| +4 | 7 | 0.83 | 0.94 | 1.06 |
| Room temperature | 1 | 0.81 | 0.91 | 1.02 |
| Room temperature | 7 | 0.88 | 0.94 | 1.01 |

GM**:** Geometric Mean potency derived from 6 estimates in all cases; LCL and UCL: Lower and Upper 95% confidence limits

**Supplemental Table 10: Summary of results from freeze-thaw studies of candidate preparation 17/236 using an L929 cell cytotoxicity assay**

| Number of freeze/thaw cycles | LCL | Relative Potency to a freshly reconstituted ampoule | UCL |
| --- | --- | --- | --- |
| 1x | 0.90 | 0.97 | 1.05 |
| 2x | 0.96 | 1.04 | 1.14 |
| 3x | 0.98 | 1.07 | 1.16 |
| 4x | 0.93 | 0.99 | 1.06 |

GM: Geometric Mean potency derived from 9 estimates in all cases; LCL and UCL: Lower and Upper 95% confidence limits

**Supplemental Table 11: Summary of results from accelerated temperature degradation studies of candidate preparation 17/236 assayed using an L929 cell cytotoxicity assay**

| Time stored (years) | Storage Temperature (°C) | LCL | Relative Potency^1^ to -70°C | UCL |
| --- | --- | --- | --- | --- |
| 1.208 | -20 | 0.93 | 0.96 | 0.99 |
| 1.208 | +4 | 0.96 | 1.00 | 1.05 |
| 1.208 | +20 | 0.97 | 1.00 | 1.03 |
| 1.208 | +37 | 0.97 | 1.02 | 1.06 |
| 1.208 | +45 | 1.03 | 1.09 | 1.17 |

GM**:** Geometric Mean potency derived from 9 estimates in all cases; LCL and UCL: Lower and Upper 95% confidence

**Supplemental Table 12: Laboratory geometric mean content estimates (μg/ml) and inter-laboratory GCV values for spiked samples S1-S24 calculated relative to kit or in-house standards**

| Sample | Laboratory | | | | | | | | | | | | | | Overall GM | Theoretical amount^1^ | Inter-lab GCV% |
| --- | --- | --- | --- | --- | --- | --- | --- | --- | --- | --- | --- | --- | --- | --- | --- | --- | --- |
|  | 1Ta | 1Tb | 2T | 3T | 4T | 6T | 7T | 8T | 9T | 10T | 12T | 14T | 15T | 16T |  |  |  |
| S1 | bl | bl | bl | bl | bl | bl | bl | bl | bl | bl | bl | bl | bl | bl |  | 0 |  |
| S2 | 1.86 | 1.93 | 1.67 | 2.45 | 2.09 | 1.88 | 2.22 | 1.91 | 1.83 | 1.63 | 2.43 | 2.63 | 2.17 | 1.74 | 2.01 | 2 | 16.04 |
| S3 | 5.43 | 5.20 | 5.18 | 6.47 | 5.84 | 6.03 | 5.61 | 5.98 | 5.59 | 4.74 | 7.07 | 7.57 | 5.87 | 5.70 | 5.83 | 6 | 13.08 |
| S4 | 12.3 | 10.8 | 10.5 | 12.3 | 12.2 | 10.8 | 10.6 | 12.2 | 11.2 | 9.12 | 12.7 | 13.7 | 12.0 | 9.89 | 11.40 | 12 | 11.70 |
| S5 | bl | bl | bl | bl | bl | bl | bl | bl | bl | bl | bl | bl | bl | bl |  | 0 |  |
| S6 | 2.06 | 1.87 | 1.80 | 2.57 | 2.08 | 1.57 | 1.67 | 1.73 | 1.76 | 1.61 | 2.41 | 2.70 | 2.16 | 1.86 | 1.96 | 2 | 18.76 |
| S7 | 6.36 | 5.97 | 5.20 | 7.25 | 5.58 | 4.34 | 5.77 | 5.85 | 5.43 | 4.58 | 6.66 | 7.60 | 6.29 | 5.49 | 5.82 | 6 | 16.93 |
| S8 | 11.2 | 12.2 | 11.7 | 13.8 | 13.2 | 8.66 | 11.8 | 11.3 | 10.4 | 8.90 | 12.5 | 14.0 | 12.5 | 11.9 | 11.61 | 12 | 15.34 |
| S9 | bl | bl | bl | bl | bl | bl | bl | bl | bl | bl | bl | bl | bl | bl |  | 0 |  |
| S10 | 1.96 | 1.86 | 2.09 | 2.39 | 2.06 | 2.24 | 1.96 | 1.64 | 1.67 | 1.65 | 2.21 | 2.50 | 2.14 | 1.74 | 1.99 | 2 | 14.82 |
| S11 | 6.30 | 5.72 | 5.86 | 6.83 | 5.91 | 6.32 | 5.57 | 5.93 | 5.14 | 4.76 | 6.76 | 7.46 | 6.47 | 5.28 | 5.98 | 6 | 12.91 |
| S12 | 12.3 | 11.2 | 9.71 | 12.3 | 12.7 | 10.9 | 11.1 | 11.2 | 9.73 | 9.74 | 12.5 | 14.9 | 13.2 | 7.75 | 11.22 | 12 | 17.75 |
| S13 | bl | bl | bl | bl | bl | bl | bl | bl | bl | bl | bl | bl | bl | bl |  | 0 |  |
| S14 | 1.99 | 2.06 | 1.94 | 2.46 | 2.07 | 1.59 | 1.96 | 1.68 | 1.66 | 1.53 | 2.33 | 2.58 | 1.97 | 1.95 | 1.96 | 2 | 16.97 |
| S15 | 6.21 | 6.07 | 5.47 | 7.03 | 5.87 | 4.46 | 5.78 | 5.73 | 5.20 | 4.79 | 6.78 | 7.51 | 5.94 | 5.24 | 5.81 | 6 | 15.40 |
| S16 | 11.7 | 12.6 | 10.6 | 13.6 | 12.0 | 8.76 | 11.9 | 10.4 | 11.3 | 9.50 | 12.1 | 14.1 | 11.3 | 9.59 | 11.29 | 12 | 14.57 |
| S17 | 1.83 | 2.03 | 1.96 | 2.47 | 2.09 | 2.16 | 2.07 | 1.66 | 1.67 | 1.55 | 2.30 | 2.88 | 2.20 | 1.97 | 2.03 | 2 | 17.93 |
| S18 | 4.72 | 5.43 | 4.51 | 5.82 | 4.44 | 4.39 | 4.97 | 4.24 | 4.16 | 4.23 | 5.56 | 6.19 | 5.06 | 4.61 | 4.84 | 5 | 13.65 |
| S19 | 1.86 | 2.16 | 1.78 | 2.38 | 1.97 | 2.10 | 2.24 | 1.91 | 1.67 | 1.55 | 2.37 | 2.51 | 2.46 | 1.95 | 2.04 | 2 | 16.06 |
| S20 | 4.84 | 5.33 | 4.98 | 5.96 | 5.17 | 4.73 | 4.89 | 4.30 | 4.25 | 3.96 | 5.70 | 6.16 | 4.78 | 5.01 | 4.97 | 5 | 13.41 |
| S21 | bl | bl | bl | bl | bl | bl | bl | bl | bl | bl | bl | bl | bl | bl |  | 0 (+ADA) |  |
| S22 | 1.76 | 1.76 | 1.38 | 2.04 | 1.49 | 1.39 | 1.69 | 1.30 | 1.46 | 1.35 | 2.06 | 2.28 | 1.52 | 1.48 | 1.62 | 2 (+ADA) | 19.27 |
| S23 | 5.28 | 6.09 | 5.04 | 6.80 | 4.60 | 4.25 | 5.48 | 5.14 | 5.59 | 4.70 | 6.55 | 7.18 | 5.62 | 5.17 | 5.48 | 6 (+ADA) | 16.36 |
| S24 | 11.7 | 12.0 | 10.3 | 13.0 | 11.7 | 8.93 | 10.3 | 10.8 | 9.93 | 9.75 | 12.6 | 13.5 | 11.7 | 11.3 | 11.16 | 12 (+ADA) | 12.51 |

GM: Geometric Mean; GCV: Geometric Coefficient of Variation (%); bl : below limit of quantitation; loq : level at limit of quantitation; ADA : anti-drug antibodies; shaded boxes : unspiked samples or spiked but containing ADA; ^1^ : adalimumab

| Sample | Laboratory | | | | | | | | | | | | | | Overall GM | Theoretical amount^1^ | Inter-lab GCV |
| --- | --- | --- | --- | --- | --- | --- | --- | --- | --- | --- | --- | --- | --- | --- | --- | --- | --- |
|  | 1Ta | 1Tb | 2T | 3T | 4T | 6T | 7T | 8T | 9T | 10T | 12T | 14T | 15T | 16T |  |  |  |
| S1 | bl | loq | bl | bl | bl | bl | bl | bl | bl | bl | bl | bl | bl | bl |  | 0 |  |
| S2 | 2.26 | 2.51 | 1.64 | 2.43 | 2.14 | 2.30 | 2.87 | 2.17 | 2.02 | 1.96 | 3.30 | 2.63 | 2.19 | 2.38 | 2.31 | 2 | 18.62 |
| S3 | 6.28 | 6.34 | 4.91 | 6.36 | 5.62 | 6.83 | 6.81 | 6.65 | 5.79 | 5.25 | 7.50 | 7.01 | 5.79 | 6.83 | 6.24 | 6 | 12.69 |
| S4 | 15.1 | 12.7 | 9.63 | 12.3 | 11.8 | 13.0 | 12.3 | 13.3 | 11.5 | 10.4 | 12.9 | 12.5 | 11.8 | 10.8 | 12.08 | 12 | 11.97 |
| S5 | bl | loq | bl | bl | bl | bl | bl | bl | bl | bl | bl | bl | bl | bl |  | 0 |  |
| S6 | 2.51 | 2.46 | 1.76 | 2.55 | 2.13 | 1.93 | 2.20 | 1.97 | 1.91 | 1.92 | 3.25 | 2.70 | 2.19 | 2.23 | 2.23 | 2 | 18.11 |
| S7 | 7.31 | 7.20 | 4.92 | 7.13 | 5.38 | 5.06 | 6.98 | 6.52 | 5.61 | 5.10 | 7.18 | 7.04 | 6.22 | 6.61 | 6.24 | 6 | 16.07 |
| S8 | 13.5 | 14.3 | 11.0 | 13.9 | 12.7 | 10.6 | 13.7 | 12.3 | 11.6 | 10.1 | 12.6 | 12.8 | 12.4 | 13.2 | 12.41 | 12 | 11.04 |
| S9 | bl | bl | bl | bl | bl | bl | bl | bl | bl | bl | bl | bl | bl | bl |  | 0 |  |
| S10 | 2.38 | 2.48 | 2.04 | 2.37 | 2.12 | 2.67 | 2.55 | 1.87 | 1.90 | 1.96 | 3.09 | 2.52 | 2.19 | 2.31 | 2.30 | 2 | 15.36 |
| S11 | 7.28 | 6.91 | 5.52 | 6.71 | 5.68 | 7.05 | 6.75 | 6.60 | 5.55 | 5.28 | 7.31 | 6.92 | 6.35 | 6.51 | 6.42 | 6 | 11.55 |
| S12 | 15.2 | 13.1 | 9.06 | 12.3 | 12.2 | 13.0 | 12.8 | 12.2 | 10.7 | 11.2 | 12.8 | 13.7 | 13.0 | 9.36 | 12.08 | 12 | 15.28 |
| S13 | bl | loq | bl | bl | bl | bl | bl | bl | bl | bl | bl | bl | bl | bl |  | 0 |  |
| S14 | 2.43 | 2.67 | 1.90 | 2.44 | 2.13 | 1.93 | 2.55 | 1.91 | 1.93 | 1.86 | 3.17 | 2.59 | 1.99 | 2.60 | 2.26 | 2 | 18.47 |
| S15 | 7.13 | 7.29 | 5.16 | 6.91 | 5.65 | 5.09 | 6.99 | 6.38 | 5.46 | 5.29 | 7.29 | 6.97 | 5.85 | 6.44 | 6.23 | 6 | 14.55 |
| S16 | 14.3 | 14.8 | 9.44 | 13.7 | 11.6 | 10.6 | 13.8 | 11.3 | 11.7 | 10.9 | 12.3 | 12.9 | 11.1 | 10.9 | 11.99 | 12 | 13.91 |
| S17 | 2.26 | 2.67 | 1.92 | 2.45 | 2.15 | 2.78 | 2.69 | 1.89 | 1.71 | 1.89 | 3.20 | 2.86 | 2.24 | 2.56 | 2.34 | 2 | 20.53 |
| S18 | 5.50 | 6.59 | 4.30 | 5.71 | 4.33 | 5.40 | 6.07 | 4.76 | 4.30 | 4.71 | 6.26 | 5.82 | 5.05 | 5.82 | 5.28 | 5 | 15.63 |
| S19 | 2.27 | 2.79 | 1.74 | 2.37 | 2.03 | 2.71 | 2.89 | 2.17 | 1.79 | 1.84 | 3.24 | 2.53 | 2.48 | 2.74 | 2.36 | 2 | 21.15 |
| S20 | 5.63 | 6.48 | 4.73 | 5.84 | 5.00 | 5.78 | 5.99 | 4.83 | 4.38 | 4.40 | 6.38 | 5.79 | 4.79 | 6.20 | 5.40 | 5 | 14.70 |
| S21 | bl | bl | bl | bl | bl | bl | bl | bl | bl | bl | bl | bl | bl | bl |  | 0 (+ADA) |  |
| S22 | 2.14 | 2.30 | 1.36 | 2.03 | 1.54 | 1.83 | 2.22 | 1.49 | 1.63 | 1.64 | 2.89 | 2.30 | 1.52 | 1.86 | 1.87 | 2 (+ADA) | 24.05 |
| S23 | 6.14 | 7.32 | 4.78 | 6.67 | 4.47 | 5.24 | 6.65 | 5.75 | 6.00 | 5.15 | 7.11 | 6.46 | 5.58 | 6.36 | 5.92 | 6 (+ADA) | 15.93 |
| S24 | 14.2 | 14.0 | 9.57 | 13.1 | 11.3 | 11.6 | 12.0 | 11.8 | 10.9 | 11.2 | 12.7 | 12.3 | 11.5 | 12.4 | 11.99 | 12 (+ADA) | 10.82 |

**Supplemental Table 13: Laboratory geometric mean content estimates (μg/ml) and inter-laboratory GCV values for spiked samples S1-S24 calculated relative to sample A**

GM: Geometric Mean; GCV: Geometric Coefficient of Variation (%); bl : below limit of quantitation; loq : level at limit of quantitation; ADA : anti-drug antibodies; shaded boxes : unspiked samples or spiked but containing ADA; ^1^ : adalimumab
